# Neto1 Is a Novel CUB-Domain NMDA Receptor–Interacting Protein Required for Synaptic Plasticity and Learning

**DOI:** 10.1371/journal.pbio.1000041

**Published:** 2009-02-24

**Authors:** David Ng, Graham M Pitcher, Rachel K Szilard, Andréa Sertié, Marijana Kanisek, Steven J Clapcote, Tatiana Lipina, Lorraine V Kalia, Daisy Joo, Colin McKerlie, Miguel Cortez, John C Roder, Michael W Salter, Roderick R McInnes

**Affiliations:** 1 Program in Developmental Biology, The Research Institute, Hospital for Sick Children, Toronto, Ontario, Canada; 2 Program in Genetics & Genome Biology, The Research Institute, Hospital for Sick Children, Toronto, Ontario, Canada; 3 Department of Molecular Genetics, University of Toronto, Toronto, Ontario, Canada; 4 Neurosciences & Mental Health, The Research Institute, Hospital for Sick Children, Toronto, Ontario, Canada; 5 Department of Physiology, University of Toronto, Toronto, Ontario, Canada; 6 Mount Sinai Hospital Research Institute, Toronto, Ontario, Canada; 7 Institute of Medical Science, University of Toronto, Toronto, Ontario, Canada; 8 Department of Laboratory Medicine & Pathobiology, University of Toronto, Toronto, Ontario, Canada; 9 Department of Pediatrics, University of Toronto, Toronto, Ontario, Canada; Mount Sinai School of Medicine, United States of America

## Abstract

The N-methyl-D-aspartate receptor (NMDAR), a major excitatory ligand-gated ion channel in the central nervous system (CNS), is a principal mediator of synaptic plasticity. Here we report that neuropilin tolloid-like 1 (Neto1), a complement C1r/C1s, Uegf, Bmp1 (CUB) domain-containing transmembrane protein, is a novel component of the NMDAR complex critical for maintaining the abundance of NR2A-containing NMDARs in the postsynaptic density. Neto1-null mice have depressed long-term potentiation (LTP) at Schaffer collateral-CA1 synapses, with the subunit dependency of LTP induction switching from the normal predominance of NR2A- to NR2B-NMDARs. NMDAR-dependent spatial learning and memory is depressed in Neto1-null mice, indicating that Neto1 regulates NMDA receptor-dependent synaptic plasticity and cognition. Remarkably, we also found that the deficits in LTP, learning, and memory in Neto1-null mice were rescued by the ampakine CX546 at doses without effect in wild-type. Together, our results establish the principle that auxiliary proteins are required for the normal abundance of NMDAR subunits at synapses, and demonstrate that an inherited learning defect can be rescued pharmacologically, a finding with therapeutic implications for humans.

## Author Summary

The fundamental unit for information processing in the brain is the synapse, a highly specialized site of communication between the brain's multitude of individual neurons. The strength of the communication at each synapse changes in response to neuronal activity—a process called synaptic plasticity—allowing networks of neurons to adapt and learn. How synaptic plasticity occurs is a major question in neurobiology. A central player in synaptic plasticity is an assembly of synaptic proteins called the NMDA receptor complex. Here, we discovered that the protein Neto1 is a component of the NMDA receptor complex. Neto1-deficient mice had a dramatic decrease in the number of NMDA receptors at synapses and consequently, synaptic plasticity and learning were impaired. By indirectly enhancing the function of the residual NMDA receptors in Neto1-deficient mice with a small molecule, we restored synaptic plasticity and learning to normal levels. Our findings establish the principle that inherited abnormalities of synaptic plasticity and learning due to NMDA receptor dysfunction can be pharmacologically corrected. Our discoveries also suggest that synaptic proteins that share a molecular signature, called the CUB domain, with Neto1 may be important components of synaptic receptors across species, because several CUB-domain proteins in worms have also been found to regulate synaptic receptors.

## Abstract 3

Spatial learning and memory depend on the N-methyl-D-aspartic acid receptor, a synaptic ion channel regulated by Neto1. Impaired cognition due to the absence of Neto1 can be rescued pharmacologically, a finding with implications for the therapy of inherited learning defects in humans.

## Introduction

In the mammalian central nervous system, excitatory transmission at synapses is mediated primarily by the amino acid glutamate, acting through the postsynaptic α-amino-3-hydroxy-5-methyl-4-isoxazolepropionic acid receptors (AMPARs) and N-methyl-D-aspartic acid receptors (NMDARs) [1]. Basal synaptic transmission is principally mediated by AMPARs, which are rapidly activated by glutamate, while the more slowly activated NMDAR primarily mediates various forms of synaptic plasticity. A large body of evidence indicates the NMDAR is essential for a prominent form of synaptic plasticity, long-term potentiation (LTP) at Schaffer collateral-CA1 synapses, and for hippocampal-dependent spatial learning and memory [2,3].

The core NMDAR is a heterotetramer comprised of two obligate NR1 subunits and two NR2(A-D) subunits [1]. These core subunits are embedded in a multiprotein complex that includes more than 70 NMDAR-associated proteins [4]. An emerging theme in NMDAR biology is that proteins associated with the core NMDAR may have important roles in the trafficking, stability, subunit composition, or function of NMDARs and may therefore be critical for synaptic plasticity, learning, and memory [5]. However, proteins that function to specifically maintain synaptic NMDARs, which are well-known for AMPARs, have been elusive for NMDARs.

We investigated the complement C1r/C1s, Uegf, Bmp1 (CUB) domain protein neuropilin tolloid-like 1 (Neto1) [6,7], which we have discovered to be an NMDAR-associated protein [8]. The CUB domain is an extracellular motif of approximately 110 amino acids originally identified in the complement subunits Clr/Cls, sea urchin epidermal growth factor, and bone morphogenetic protein 1 (BMP1). Comprised of 10 β-strands forming a “jellyroll” topology [9], CUB domains mediate protein-protein interactions [10]. Notably, the CUB domain protein SOL-1 in Caenorhabditis elegans has been shown to be a component of the GLR-1 glutamate receptor [11], required for its gating [12], and another C. elegans CUB-domain protein LEV-10 has been found to regulate the clustering of acetylcholine receptors at the neuromuscular junction [13]. Whether CUB domain proteins are significant components or regulators of neurotransmitter receptor complexes at vertebrate synapses is unknown despite the presence of ∼100 identified or predicted CUB domain proteins in the vertebrate genome [14].

To investigate the role of Neto1 in the biology of mammalian excitatory synapses, we determined the molecular basis of the Neto1:NMDAR interaction and defined the nonredundant functions of Neto1 in synaptic plasticity, learning, and memory using Neto1 protein null mice. We found that Neto1 interacts with the core NMDAR subunits, NR2A and NR2B, and with the scaffolding protein postsynaptic density-95 (PSD-95). The complete loss of Neto1 reduced the abundance of NR2A but not NR2B subunits in the PSD of the hippocampus, leading to a decrease in the amplitude of synaptic NMDAR currents and a switch from the normal predominance of NR2A- to NR2B-containing NMDARs at Schaffer collateral-CA1 synapses. In Neto1-null mice, LTP at these synapses was reduced and spatial learning and memory was impaired. By indirectly enhancing NMDAR synaptic currents in the Neto1-null mice using the ampakine CX546 [15], we rescued the deficits in both LTP and spatial learning and memory.

## Results

### Neto1 Is a Synaptic Transmembrane Protein

**Figure 1:**
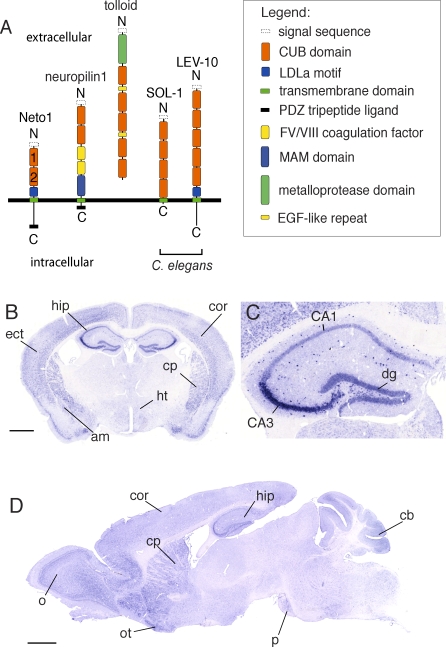
Neto1, a CUB-Domain Transmembrane Protein Expressed in the Brain (A) Domain organization of the predominant isoform of Neto1 and related CUB-domain proteins. (B–D) In situ hybridization for *Neto1* mRNA in adult wild-type brain sections. (B) Coronal. (C) Enlarged region of hippocampus. (D) Sagittal. am, amygdala; CA1 and CA3, pyramidal neurons of Cornu Ammonis regions 1 and 3; cb, cerebellum; cor, cerebral cortex; cp, caudate-putamen; dg, dentate gyrus; ect, entorhinal cortex; hip, hippocampus; ht, hypothalamus; o, olfactory bulb; ot, olfactory tubercle; p, pons. Scale bar: 1 mm.

Neto1 encodes a 533 amino acid polypeptide with an N-terminal ER signal sequence, two CUB domains, one low-density lipoprotein receptor domain class A (LDLa) motif, a transmembrane domain, and a cytoplasmic tail terminating in a class I PDZ binding tripeptide ligand (TRV-COOH) (Figure 1A). We designated this protein neuropilin tolloid-like 1 (Neto1) [8], because the first CUB domain is most similar (∼40% identity) to the CUB domains of neuropilins [16,17] and tolloid [18].

To elucidate the role of Neto1 in the brain, we first examined the expression pattern of its mRNA. In adult mice, *Neto1* mRNA was present throughout the central nervous system (Figure 1B–1D and Figure S1), with strong expression in cerebral cortex, hippocampus, olfactory bulb, olfactory tubercle, and caudate putamen.

**Figure 2:**
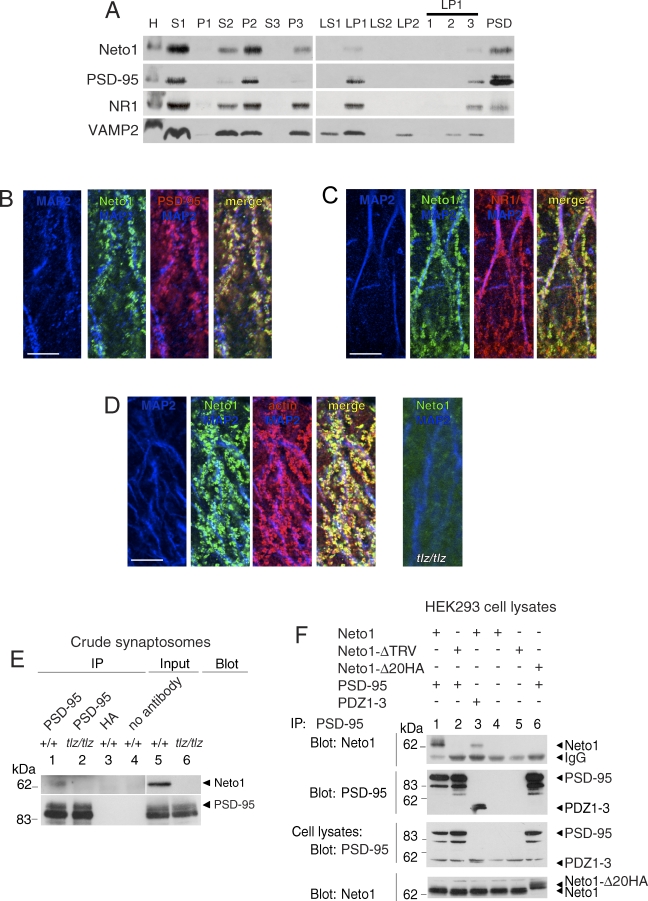
Neto1 is a PSD Protein Localized to Dendritic Spines and Interacts with PSD-95 through a C-Terminal PDZ Tripeptide (A) Subcellular fractionation profile of Neto1, PSD-95, NR1, and VAMP2. H, homogenate; LP1, synaptosomal membrane fraction; LP1–1, LP1–2 (synaptic plasma membranes), and LP1–3 designate the bands located at the interfaces of the 15%–25%, 25%–35%, and 35%–45% sucrose solutions, respectively; LP2, crude synaptic vesicle fraction; LS1, supernatant above LP1; LS2, supernatant above LP2; P1, nuclei and cell debris; P2, crude synaptosomal fraction; P3, light membrane fraction; PSD, postsynaptic density fraction. S1, supernatant above P1; S2, supernatant above P2; S3, cytosolic fraction; Equal amounts of protein from cellular fractions were loaded, except for lane PSD, where 4 μg of protein was loaded. (B–D) Confocal micrographs of immunostained wild-type or Neto1-null (*tlz/tlz*) hippocampus. Scale bar, 5 μm. (E) Immunoblots of immunoprecipitates from wild-type (+/+) and Neto1-null crude synaptosomes. As a negative control, anti-hemagglutinin antibody (HA) did not immunoprecipitate either Neto1 or PSD-95. Note that the protein detected by anti-Neto1 antibody was not observed in crude synaptosomes or immunoprecipitates from Neto1-null mice, demonstrating the specificity of the anti-Neto1 antibody. For blots probed with Neto1 antibody, the exposure time for lanes 1–4 was ∼ten times more than for lanes 5 and 6. Blot, antibodies used for immunoblot analysis; IP, antibodies used for immunoprecipitation; Input, crude synaptosomal protein. (F) Immunoblot of immunoprecipitates from transfected HEK293 cell lysates. The identities of the transfected cDNAs are indicated above each lane. Neto1 and PSD-95 are full-length proteins. The Neto1-ΔTRV protein lacks the C-terminal PDZ ligand tripeptide TRV; Neto1-Δ20HA is a deletion construct in which the C-terminal 20 amino acid residues are replaced by two copies of the HA epitope tag; PDZ1–3 is a truncated PSD-95 protein composed of only the PDZ1, 2, and 3 domains. Similar results were observed in each of three experiments.

To identify the subcellular compartments in which Neto1 is localized, we performed subcellular fractionation and immunoblotting experiments of whole mouse brain lysates. Because the C-terminal sequence of Neto1 suggested that it localized to the PSD (see below), we employed a cell fractionation strategy that separated synaptic subcompartments [19]. Neto1 was prominently expressed in the crude synaptosomal (Figure 2A, lane S2) and PSD fractions, but was absent from the synaptic vesicle fraction (Figure 2A, lane LP2). To visualize the cellular distribution of Neto1, we examined immunostained hippocampal sections by confocal microscopy. We found that Neto1 immunostaining decorated MAP2 positive dendritic arbors and co-localized with that of PSD-95 (Figure 2B) and NR1 (Figure 2C). We also found that Neto1 co-localized with actin (Figure 2D), which is highly enriched in dendritic spines in the hippocampus [20]. The immunostaining for Neto1 was not detected in hippocampus from Neto1-null (*Neto1^tlz/tlz^*, see below) mice (Figure 2D, right), indicating that the staining was not nonspecific. Together, these findings demonstrate that Neto1 is a component of the PSD of excitatory synapses.

### Neto1 Binds to PSD-95 through PDZ Ligand-PDZ Domain Interactions

**Figure 3:**
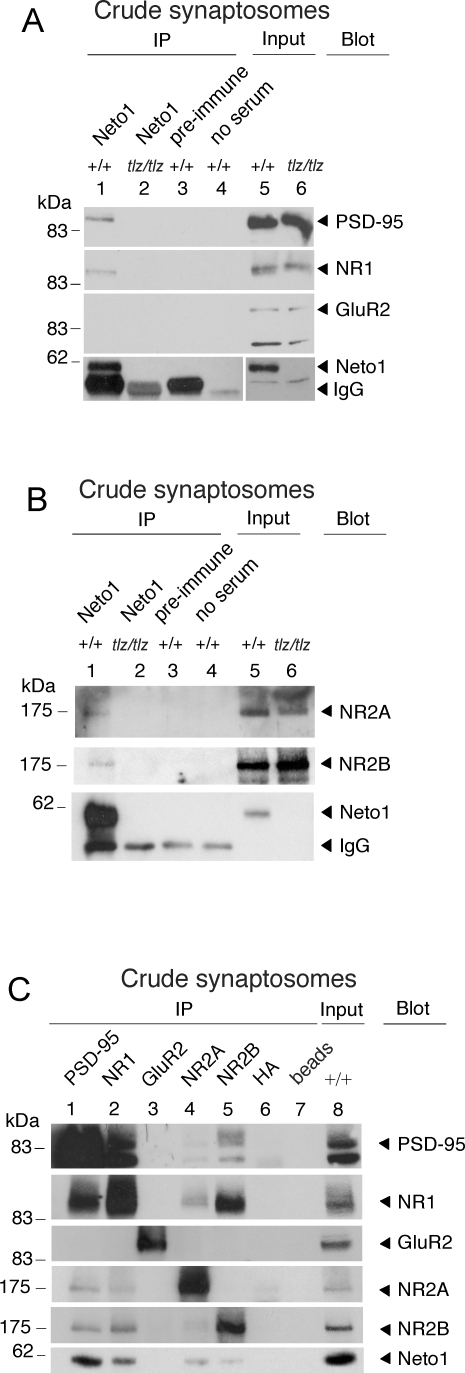
Neto1 Associates with NMDARs In Vivo (A–C) Immunoblots of immunoprecipitates from adult wild-type (+/+) and Neto1-null (*tlz/tlz*) crude synaptosomes. IgG, immunoglobulin. For blots probed with Neto1 antibody in (A), the exposure time for lanes 1–4 was ∼ten times less than for lanes 5 and 6. Blot, antibody used for immunoblot analysis; IP, antibody used for immunoprecipitation. Similar results were observed in each of three experiments.

The sequence of the C-terminal tripeptide of Neto1, TRV, suggested that it is a PDZ ligand, predicted to bind preferentially to the third PDZ domain (PDZ3) of PSD-95 [21,22]. Using the yeast two-hybrid system, we established that the cytoplasmic domain of Neto1 (Neto1-cd) associated with the full-length PDZ proteins PSD-95 (Figure S2), PSD-93, and SAP102, but not with SAP-97 or NIP [23,24] (unpublished data). Furthermore, using crude synaptosomal fractions, we determined that anti-PSD-95 antibodies co-immunoprecipitated Neto1 from wild-type (*Neto1^+/+^*) mouse brain (Figure 2E). Conversely, anti-Neto1 antibody co-immunoprecipitated PSD-95 from *Neto1^+/+^* (Figure 3A, lane 1) but not from Neto1-null crude synaptosomal fractions (Figure 3A, lane 2). Negative control antibodies did not immunoprecipitate either Neto1 or PSD-95 (Figures 2E and 3A). The Neto1 cytoplasmic domain bound most strongly to PDZ3 of PSD-95, binding that was completely dependent on the C-terminal TRV of Neto1, in both the two-hybrid system (Figure S2) and in HEK293 cells (Figure 2F, lane 2). Moreover, the Neto1 cytoplasmic domain bound to a truncated PSD-95 polypeptide (PDZ1–3) containing only the three PDZ domains (Figure 2F, lane 3). Altogether, these findings indicate that Neto1 associates with PSD-95 in brain synapses through the binding of its C-terminal tripeptide with the PDZ domains of PSD-95.

### Neto1 Interacts with NMDARs

Because PSD-95 is a prominent NMDAR scaffold protein [25,26], we asked whether Neto1 associates with NMDARs. We found that anti-Neto1 antibodies co-immunoprecipitated the NR1, NR2A, and NR2B NMDAR subunits from crude synaptosomal fractions of wild-type but not Neto1-null mice (Figure 3A and 3B, lanes 1 and 2), whereas pre-immune antibodies did not (Figure 3A and 3B, lane 3). Reciprocally, anti-NR1, anti-NR2A, and anti-NR2B antibodies co-immunoprecipitated Neto1 from wild-type synaptosomal fractions (Figure 3C, lanes 2, 4, and 5, respectively). In contrast, we were unable to co-immunoprecipitate Neto1 and GluR2 (Figure 3A, lane 1 and Figure 3C, lane 3), a major subunit of the AMPAR [27]. We therefore conclude that Neto1 is a component of the NMDAR complex but is not a general component of ionotropic glutamate receptor complexes.

**Figure 4:**
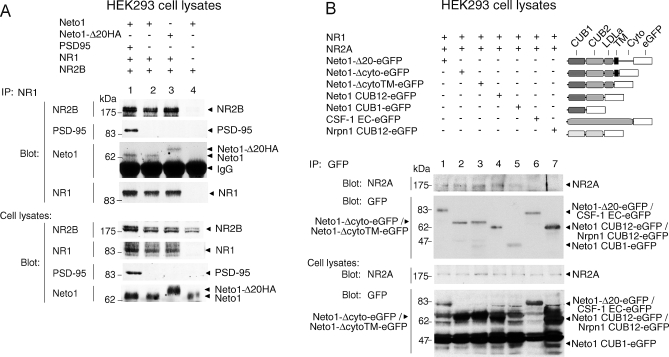
Neto1 Binds to NMDA Receptors Independently of the C-Terminal PDZ Ligand (A, B) Immunoblots of immunoprecipitates from transfected HEK293 cell lysates. The transfected cDNAs are shown above each lane. *CSF-1 EC-eGFP* encodes the extracellular domain of CSF-1 fused to eGFP. *Nrpn1 CUB12-eGFP* encodes the two CUB domains from neuropilin-1 fused to eGFP. Blot, antibody used for immunoblot analysis; IP, antibody used for immunoprecipitation.

To determine whether the association of Neto1 with NMDARs was entirely dependent upon the binding of its C-terminal PDZ ligand to PSD-95, we examined the binding of PSD-95 to an hemagglutinin (HA)-tagged Neto1 protein lacking the C-terminal 20 amino acids (Neto1-Δ20HA). As predicted both by the interaction between PSD-95 and the NR2 subunits of the NMDAR [28], and by the binding of Neto1 to PSD-95 described above, we found that Neto1 was co-immunoprecipitated by anti-NR1 antibodies from lysates of cells co-expressing Neto1, PSD-95, NR1, and NR2B (Figure 4A, lane 1). Unexpectedly, however, anti-NR1 antibodies co-immunoprecipitated Neto1-Δ20HA (Figure 4A, lane 3). Moreover, Neto1 or Neto1-Δ20HA co-immunoprecipitated with both NR1 and NR2B, even in the absence of PSD-95 (Figure 4A, lane 2 and 3, respectively). These results indicate that the binding of Neto1 to PSD-95 was not required for Neto1 to interact with the NMDAR, and that Neto1 interacts with NMDARs through a PSD-95-independent mechanism.

### The N-terminal CUB Domain of Neto1 Is Sufficient for Its Interaction with NMDARs

To identify the region of Neto1 that mediates the PSD-95-independent association between Neto1 and NMDARs, we examined the ability of a series of C-terminally deleted Neto1 proteins to co-immunoprecipitate with NMDARs from HEK293 cell lysates. Removal of the cytoplasmic tail and transmembrane domain of Neto1 did not abolish the Neto1:NMDAR interaction (Figure 4B, lanes 2 and 3), suggesting that it was mediated by the ectodomain of Neto1. Moreover, a construct expressing only the signal sequence and N-terminal CUB domain of Neto1 was sufficient to mediate the NMDAR association (Figure 4B, lane 5). In contrast, no binding was observed between NMDARs and the ectodomain of CSF-1 (Figure 4B, lane 6), or between NMDARs and the CUB domains of neuropilin-1 (Figure 4B, lane 7). These results indicate that the Neto1:NMDAR extracellular interaction is dependent on the first CUB domain of Neto1.

### Neto1 Interacts with NR2 but Not NR1 Subunits

**Figure 5:**
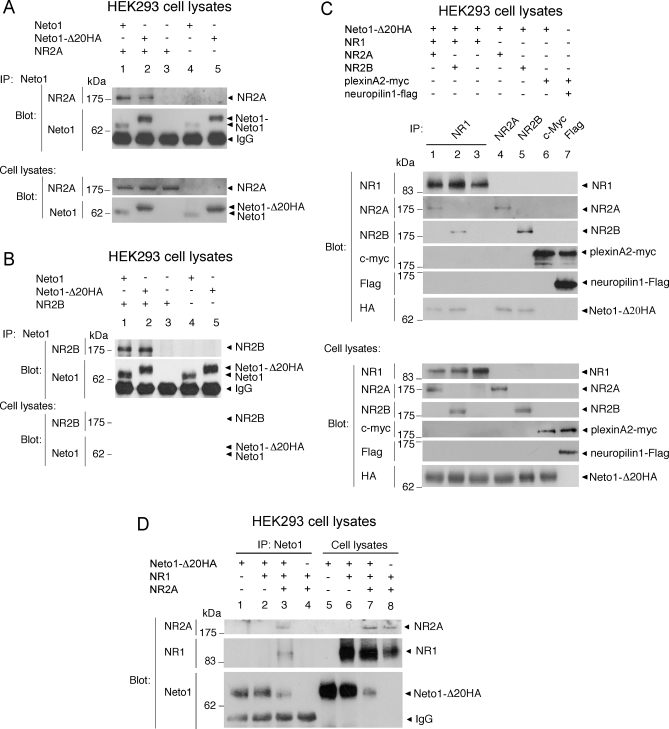
Neto1 Binds to NR2, but Not to NR1 Subunits (A–D) Immunoblots of immunoprecipitations from transfected HEK293 cell lysates. The identities of the transfected cDNAs are shown above each lane. Blot, antibodies used for immunoblot analysis; IP, antibodies used for immunoprecipitation. Plexin-A2 and neuropilin-1 were used as a positive control for co-immunoprecipitation [74]. Similar results were observed in each of three experiments.

We next asked which NMDAR subunit mediates the Neto1:NMDAR interaction, using heterologously expressed proteins in HEK293 cells. Full-length Neto1 or Neto1-Δ20HA co-immunoprecipitated with both NR2A (Figure 5A, lanes 1 and 2, and Figure 5C, lane 4) and NR2B (Figure 5B, lanes 1 and 2, and Figure 5C, lane 5) expressed in the absence of NR1 and PSD-95. In contrast, in the absence of NR2, no association was observed between Neto1-Δ20HA and NR1 (Figure 5C, lane 3; Figure 5D, lane 2). Consequently, we conclude that the PSD-95-independent Neto1:NMDAR interaction is mediated through NR2 subunits, and that the first extracellular CUB domain of Neto1 is sufficient for this binding. The simplest model consistent with our findings is that Neto1 interacts with the NMDAR bivalently, with one Neto1:NMDAR interaction mediated through the binding of the C-terminal tripeptide of Neto1 to PSD-95, and the second through the extracellular domains of Neto1 and NR2 subunits.

### Generation of Neto1-Null Mice

**Figure 6:**
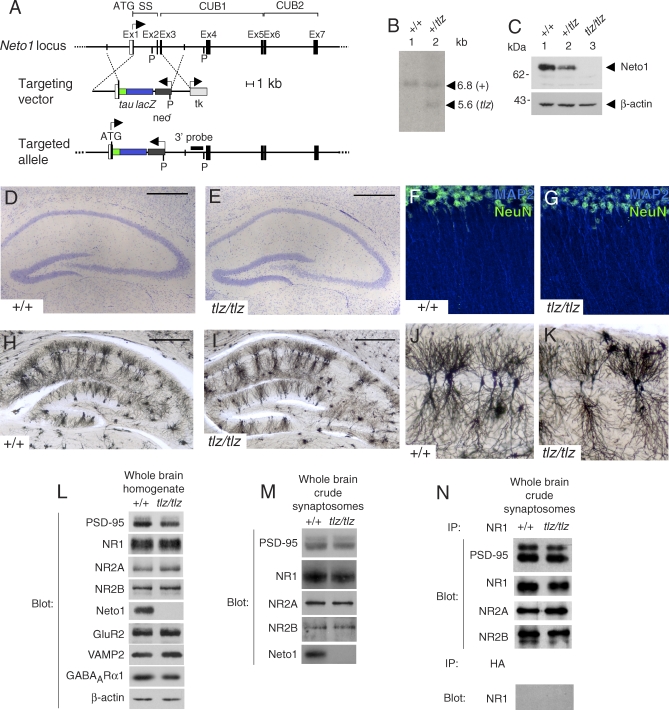
Neto1-Null Mice Have Normal Hippocampal Morphology and Express Normal Levels of Synaptic Proteins in Whole Brain (A) Upper: A portion of the *Neto1* gene showing exons (Ex). Top, encoded motifs. SS, signal sequence; open box, noncoding sequences; solid boxes, coding sequences. P, PstI restriction enzyme site. Middle: *Neto1* targeting construct. *tau-lacZ* is a reporter gene encoding a tau-β-galactosidase fusion protein. tk, thymidine kinase negative selection cassette. Lower: Targeted *Neto1^tlz/tlz^* allele after homologous recombination. Arrows indicate direction of transcription. The 3′ external probe is shown by a black rectangle. (B) Genomic Southern blot from ES cell clones digested with PstI and hybridized with the 3′ probe. (C) Immunoblot of brain lysates from *Neto1^+/+^*, *Neto1* ^+/tlz^, and Neto1-null mice using anti-Neto1 antibodies raised to the C-terminal 86 amino acids of Neto1. The arrowhead indicates the specific Neto1 immunoreactive band of ∼66 kDa. This band corresponds to glycosylated Neto1 (unpublished data). (D, E) Nissl staining of hippocampus. Scale bar, 500 μm. (F, G) Confocal micrographs of wild-type or Neto1-null (*tlz/tlz*) hippocampal slices from CA1 showing normal MAP2 and NeuN immunostaining. (H, I) Golgi staining of hippocampus. (J, K) Enlarged area of hippocampus showing Golgi-stained CA1 pyramidal neurons. (L, M) Immunoblots of different synaptic proteins from (L) whole brain and (M) crude synaptosomes. (N) Co-immunoprecipitation and immunoblotting of NMDA receptors from crude synaptosomes. Antibodies used are indicated on the left. HA; anti-hemagglutinin negative control antibody.

To determine whether Neto1 is required for normal brain function in the mouse, we disrupted the *Neto1* locus by homologous recombination in mouse embryonic stem (ES) cells. We generated a protein null allele by simultaneously introducing a *tau-lacZ* (*tlz*) reporter gene [29] in-frame into the initiation codon of the *Neto1* gene (Figure 6A–6C). Both *Neto1^+/tlz^* and Neto1-null animals were normal in overall appearance with no gross morphological abnormalities in the brain. Hematoxylin and eosin staining revealed no histological abnormalities in any brain region examined in Neto1-null mice (unpublished data), and Nissl (Figure 6D and 6E), MAP2 immunostaining (Figure 6F and 6G), and Golgi staining of the hippocampus showed no morphological defects in Neto1-null mice (Figure 6H–6K). The absence of Neto1 had no effect on the overall abundance of NR1, NR2A, NR2B, PSD-95, GluR2, VAMP2, or GABA_A_R1 proteins (Figure 6L) in whole brain extracts, or of NR1, NR2A, NR2B, or PSD-95 in crude synaptosomes (Figure 6M). Moreover, the amount of NR2A, NR2B, and PSD-95 that co-immunoprecipitated with NR1 from crude synaptosomes was normal in Neto1-null mice, indicating that the lack of Neto1 did not alter the overall abundance of the NMDAR:PSD-95 holocomplex (Figure 6N).

### Reduced Long-Term Potentiation and NMDAR Synaptic Currents in Neto1-Null Mice

**Figure 7:**
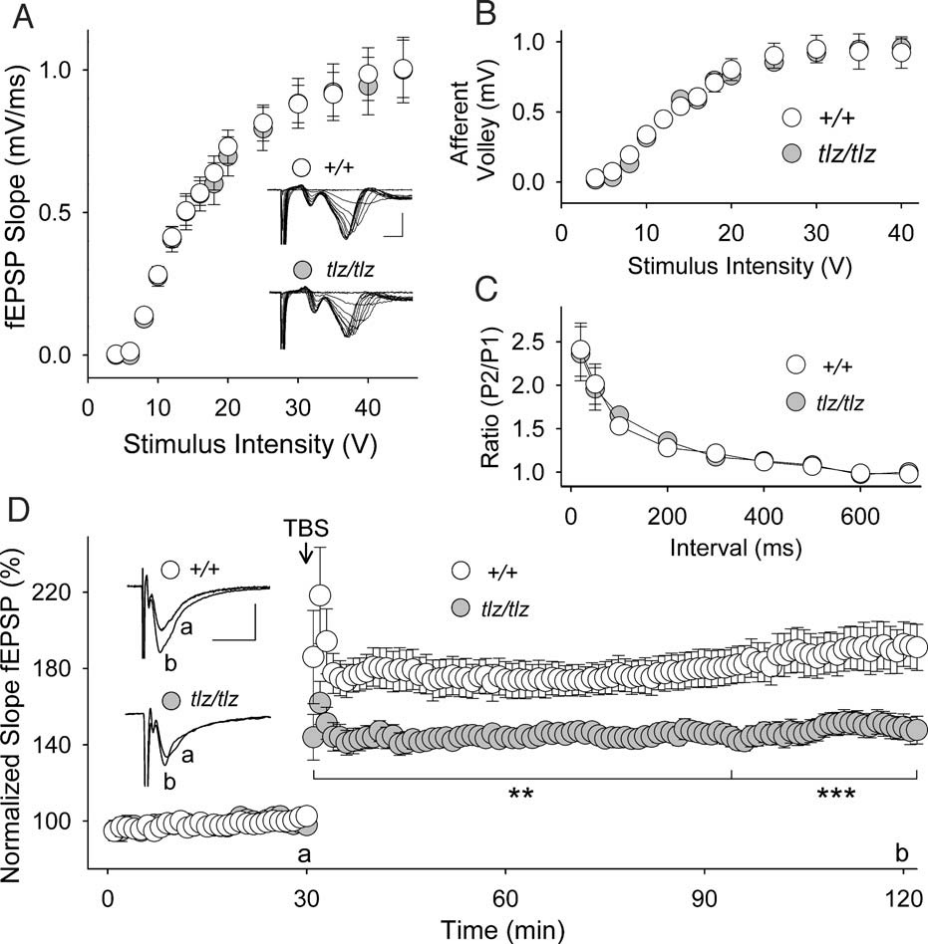
Neto1 Loss of Function Decreases tbLTP in CA1 Hippocampus (A) fEPSP slope and (B) fiber volley amplitude plotted as a function of stimulus intensity in *Neto1^+/+^* (*+/+*, open circles) and Neto1-null (*tlz/tlz*, filled circles) mice. Strength of Schaffer collateral stimulation is indicated on the horizontal axis. Representative traces show fiber volley and fEPSPs (scale bars: 2 ms, 1.5 mV). (C) Paired-pulse facilitation of fEPSPs in slices from *+/+* and *tlz/tlz* mice. Interstimulus interval is indicated on the horizontal axis. P1, fEPSP slope first response; P2, fEPSP slope second response. (D) Summary scatter plot shows grouped normalized fEPSP slope every 1 min in slices from *+/+* (*n* = 20 slices) and *tlz/tlz* (*n* = 17 slices; **, *p* < 0.01; ***, *p* < 0.001 versus *+/+*) mice. Theta-burst stimulation (TBS) was delivered to Schaffer collateral CA1 synapses at the 30-min time point. fEPSP slope was normalized to the mean slope of fEPSPs recorded during the 10-min period immediately before TBS. Inset: average of six consecutive fEPSPs recorded at the times indicated before or after theta-burst stimulation (a or b, respectively; scale bars: 15 ms, 0.4 mV). Error bars show ± standard error of the mean (SEM).

Having shown that Neto1 is a component of the NMDAR complex, we asked whether glutamatergic synaptic transmission and plasticity are altered in the absence of Neto1. Given that *Neto1* is expressed in the CA1 region of the hippocampus (Figure 1C), we studied synaptic transmission and plasticity at Schaffer collateral-CA1 synapses, which are widely used to investigate glutamatergic synaptic physiology [30]. We recorded field excitatory postsynaptic potentials (fEPSPs) in acute hippocampal slices from adult animals and used theta-burst pattern stimulation to induce long-term potentiation (tbLTP), a robust form of NMDAR-dependent synaptic plasticity [31]. Basal fEPSPs, afferent fiber volley, and paired-pulse facilitation in slices from Neto1-null mice were not different from those of wild-type littermate controls (Figure 7A–7C). In contrast, we found that tbLTP was reduced in Neto1-null mice (Figure 7D): the magnitude of the potentiation in the mutant animals was approximately 50% of that in wild-type controls 60 min and longer after theta-burst stimulation. Because paired-pulse facilitation, a measurement of presynaptic function [32], was not different in Neto1-null mice versus wild-type controls, the reduction in tbLTP is not the result of a deficit in presynaptic function. We therefore conclude that basal synaptic transmission at Schaffer collateral-CA1 synapses appears intact, whereas LTP is significantly impaired in Neto1-null mice.

**Figure 8:**
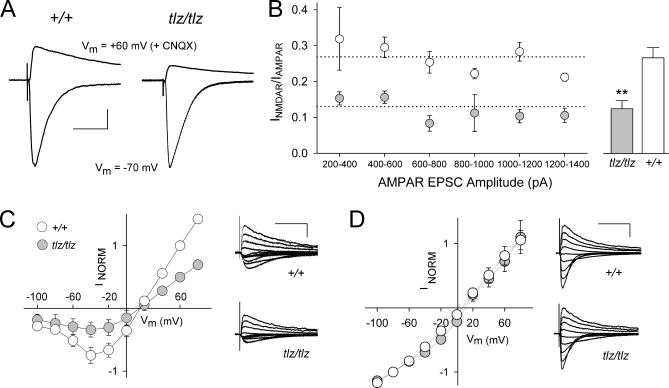
Reduction of Basal NMDAR EPSC Amplitude at Schaffer Collateral-CA1 Synapses of Neto1-Null Mice (A) Representative traces of AMPAR and NMDAR EPSCs from an individual +/+ (left) or *tlz/tlz* (right) neuron. The bottom trace in each was recorded at a holding potential of −70 mV (*V*_m_ = −70 mV) then CNQX (10 μM) was bath applied and the top traces recorded at a holding potential of +60 mV (*V*_m_ = +60 mV). Each trace is an average of six consecutive responses. For all traces the intensity of the Schaffer collateral was 10 V. I_NMDAR_/I_AMPAR_ was 0.31 for the +/+ neuron and 0.12 for the *tlz/tlz* neuron (scale bar: 80 ms, 100 pA). (B) Left: the plot shows I_NMDAR_/I_AMPAR_ as a function of AMPAR EPSC amplitude in +/+ (open circles) and *tlz/tlz* (filled circles) neurons. The range of synaptic activation was generated by an ascending series of stimulus intensities with the neuron at *V*_m_ = −70 mV and then at *V*_m_ = +60 mV + CNQX (10 μM) for each neuron tested with I_NMDAR_/I_AMPAR_ calculated for each corresponding stimulus. The data are plotted in 200 pA bins of AMPAR EPSC amplitude. The dotted line shows the overall mean of the I_NMDAR_/I_AMPAR_ for all data points across the amplitude range. Right: For each neuron, the average I_NMDAR_/I_AMPAR_ was calculated and the histogram shows the mean of I_NMDAR_/I_AMPAR_ for *tlz/tlz* (filled bar) or +/+ (open bar) neurons (**, *p* < 0.01). (C) Current-voltage (*I*-*V*) graph for pharmacologically isolated NMDARs from +/+ (open circles) and *tlz/tlz* (filled circles) mice. Right: superimposed NMDAR EPSC traces at *V*_m_ from −100 to +80 mV in steps of 20 mV (scale bars: 150 ms/ 125 pA). (D) Current-voltage (*I*-*V*) graph for AMPAR EPSCs from *Neto1^+/+^* (open circles) and Neto1-null mice (filled circles). Right: superimposed AMPAR EPSC traces (scale bars: 200 ms/ 200 pA). Error bars show ± standard error of the mean (SEM).

tbLTP at Schaffer collateral-CA1 synapses is NMDAR-dependent [31]. We investigated NMDAR excitatory postsynaptic currents (EPSCs) evoked by Schaffer collateral stimulation, by using whole-cell recordings from CA1 pyramidal neurons (Figure 8). In order to examine NMDAR EPSCs in relationship to synaptic activation, we recorded both NMDAR and AMPAR EPSCs in the same neurons in wild-type and Neto1-null slices. We found that the NMDAR:AMPAR EPSC ratio was significantly less in Neto1-null neurons (Figure 8A) regardless of the size of AMPAR EPSCs examined (Figure 8B). Because basal synaptic transmission (Figure 7) and AMPAR-EPSCs (Figure S5) in Neto1-null neurons were not different from wild-type, we interpret the decrease in NMDAR:AMPAR EPSC ratio as indicating that synaptic NMDAR currents were reduced in Neto1-null neurons. The current-voltage relationship for NMDAR EPSCs in Neto1-null mice was comparable to that of wild-type animals, demonstrating that the Mg^2+^ blockade of the NMDARs was not altered by the lack of Neto1 (Figure 8C). Furthermore, we observed no abnormalities in the current-voltage relationship for AMPARs in Neto1-null mice (Figure 8D). Thus, basal NMDAR-mediated, but not AMPAR-mediated, synaptic responses are impaired in CA1 pyramidal neurons in the absence of Neto1. These findings suggest that the impairment in NMDAR EPSCs may account for the reduced tbLTP in Neto1-null mice.

### Neto1 Is Required for the Normal Complement of Synaptic NR2A Receptors

**Figure 9:**
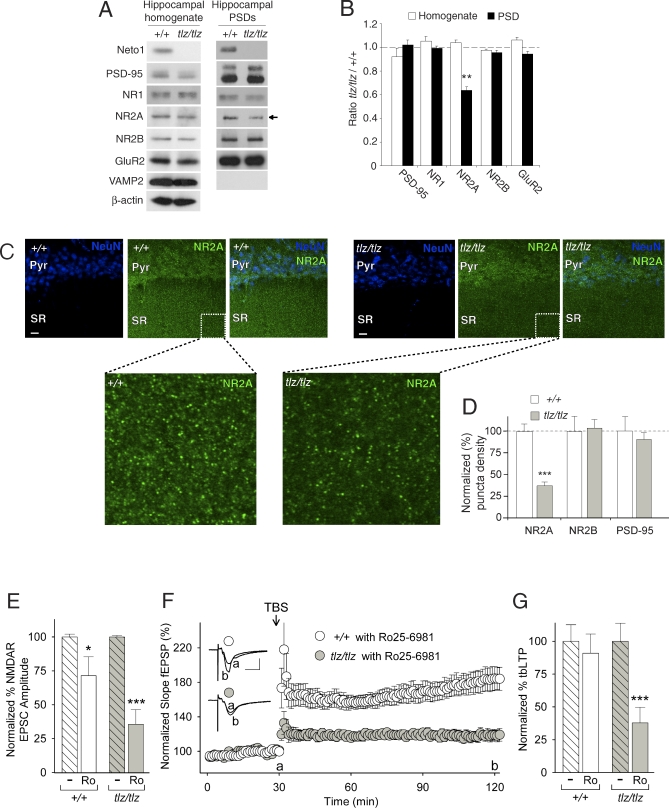
Reduction of NR2A in CA1 and tbLTP Subunit Dependency Switch from NR2A- to NR2B-NMDARs (A) Immunoblots of synaptic proteins in whole hippocampal homogenates (10 μg of protein) and the hippocampal PSD fraction (2 μg of protein) from +/+ and *tlz/tlz* mice. Antibodies used for detection are indicated at left. Blots shown are representative of four separate experiments. (B) Histogram showing normalized levels of different synaptic proteins in *tlz/tlz* hippocampal homogenate relative to that of +/+ (white bars), and *tlz/tlz* PSD fractions relative to that of +/+ (black bars). Band intensity was quantified as a mean grayscale value. **, *p* < 0.01, *t*-test, *n* = 4 pools of five pairs of hippocampi. (C) Confocal micrographs of immunostained hippocampal slices from the CA1 region. Antibodies used are indicated in each box. Scale bar, 10 μm. Pyr, pyramidal cell layer; SR; stratum radiatum. (D) Histogram of relative number of NR2A, NR2B, and PSD-95 puncta in CA1 stratum radiatum between wild-type and *Neto1*-null (*tlz/tlz*) hippocampal slices. ***, *p* < 0.005, Student's *t*-test; *n* = 3 mice/genotype. (E) Histogram of pharmacologically isolated NMDAR EPSCs from CA1 neurons in hippocampal slices from +/+ (*n =* 5 neurons) and *tlz/tlz* (*n =* 6 neurons) mice before (−) and 40 min after Ro25–6981 (Ro; 2 μM). NMDAR EPSCs were monitored every 10 s throughout the experiment; the effect of Ro25–6981 had stabilized by 30 min of application. Results are expressed as a percentage of NMDAR amplitude, with the amplitude in +/+ (white bar) and *tlz/tlz* (filled bar) slices before Ro25–6981 treatment normalized to 100%. *, *p* < 0.05 versus *Neto1^+/+^* (*+/+*) before Ro25–6981 treatment (−). ***, *p* < 0.001 versus *tlz/tlz* before Ro25–6981 treatment (−). (F) Summary scatter plot shows the grouped normalized fEPSP slope plotted every 1 min in Ro25–6981-treated slices (in ACSF beginning 30–40 min before theta-burst stimulation with a final concentration of 2 μM) from +/+ (*n* = 16 slices) and *tlz/tlz* (*n =* 9 slices) mice. Inset: average of six consecutive fEPSPs recorded at the times indicated (a or b; scale bars: 10 ms, 0.5 mV). (G) Histogram showing the theta-burst stimulation-induced increase in fEPSP slope 90 min after theta-burst stimulation in slices from *+/+* and *tlz/tlz* mice without (−) and with Ro25–6981 (Ro) treatment. Results are expressed as a percentage of theta-burst stimulation-induced increase in fEPSP slope (% tbLTP) with tbLTP in +/+ and *tlz/tlz* slices without Ro25–6981 treatment normalized to 100% (white and filled bars, respectively). ***, *p* < 0.001 versus *tlz/tlz* without Ro25–6981 treatment (−). Data are shown as mean ± standard error of the mean (SEM).

The reduction in tbLTP and NMDAR EPSCs at Schaffer collateral-CA1 synapses suggested that there might be a decrease in the abundance or function of synaptic NMDARs. We found that the abundance of NR2A in the PSD fraction from whole hippocampal lysates from Neto1-null mice was reduced by approximately one-third compared with that of wild-type littermates (Figure 9A and 9B). Consistent with this reduction, the number of NR2A puncta in stratum radiatum of the CA1 region was also reduced, by approximately 60%, in Neto1-null mice (Figure 9C and 9D). In contrast, no significant differences were observed in the abundance of PSD-95, NR1, NR2B, or GluR2 between Neto-1 null versus wild-type mice (Figure 9A and 9B). Similarly, there were no differences in the number of NR2B or PSD-95 puncta in CA1 stratum radiatum of Neto1-null mice (Figure 9D and Figure S3A and S3B). These findings indicate that Neto1 is required to establish or maintain the normal abundance of NR2A-containing NMDARs in the PSD.

To determine whether there was an overall decrease in cell surface expression of NR2A-containing NMDARs in Neto1-null mice, we quantified the abundance of biotinylated cell surface proteins in wild-type and Neto1-null hippocampal slices. No differences in the level of biotinylated NR1, NR2A, or NR2B were found in Neto1-null compared with wild-type mice (Figure S4A), indicating that the overall cell surface expression of NMDARs is normal in the hippocampus in the absence of Neto1. Similarly, total NMDA-evoked current density and the fractional current carried by NR2A-receptors were also normal in acutely isolated CA1 pyramidal neurons from Neto1-null mice (Figure S4B and S4C). Collectively, these findings indicate that lack of Neto1 does not alter the total surface expression of NMDARs, but rather decreases the targeting or stability of NR2A-containing NMDARs at synapses.

To determine whether the decreased synaptic abundance of NR2A subunits leads to a reduction in NR2A-mediated synaptic currents we examined the relative contribution of NR2A versus NR2B to NMDAR EPSCs at CA1 synapses. In the adult hippocampus, NR2A-containing NMDARs make a larger contribution to basal NMDAR-mediated synaptic transmission than those containing NR2B subunits [33]. Consequently, if the decrease in NMDAR EPSCs was due to the reduced level of NR2A-NMDARs, the relative contribution of NR2B-NMDARs to synaptic NMDAR currents would be predicted to be increased in Neto1-null mice. We therefore compared the effect of blocking NR2B-NMDARs using the NR2B-selective antagonist, Ro25–6981 [34], in wild-type and Neto1-null mice. Because Ro25–6981 is a use-dependent NMDAR blocker, we continued the regular synaptic activation (0.1 Hz) during Ro25–6981 application and calculated its effect only after NMDAR EPSCs had stabilized, 20–30 min after the start of Ro25–6981 administration. In wild-type synapses, Ro25–6981 (2 μM) reduced NMDAR EPSCs by ∼30% (Figure 9E and Figure S6). In contrast, in Neto1-null synapses the reduction was ∼70% (*p* < 0.001) (Figure 9E and Figure S6), indicating that basal NMDAR EPSCs in Neto1-null synapses are mediated primarily by NR2B-containing NMDARs. Moreover, in Neto1-null synapses, but not in those of wild-type mice, the component of the NMDAR EPSC resistant to Ro25–6981 (2 μM) decayed more rapidly than did the component sensitive to Ro25–6981 (Figure S6). Thus, the absence of Neto1 decreases the relative contribution of NR2A-containing receptors to NMDAR EPSCs at Schaffer collateral-CA1 synapses.

We investigated the impact of the decrease of synaptic NR2A-mediated currents on tbLTP at Schaffer collateral synapses. Because basal NMDAR EPSCs in Neto1-null mice were mediated primarily by NR2B-containing NMDARs, we examined the effect of blocking NR2B-NMDARs on the induction of tbLTP in wild-type and *Neto1*-null mice using Ro25–6981. In wild-type slices, Ro25–6981 (2 μM) had no effect on tbLTP (Figure 9F). In contrast, in Neto1-null slices Ro25–6981 led to a ∼60% reduction in tbLTP (Figure 9F and 9G). These findings indicate that tbLTP in Schaffer collateral-CA1 synapses of adult Neto1-null mice is mediated primarily by NR2B-containing NMDARs. Taken together, these findings demonstrate that Neto1 is required for the normal abundance of synaptic NR2A-containing NMDARs and, as a result, for the normal contribution of NR2A-NMDARs to synaptic transmission and plasticity in CA1 hippocampus.

### Impaired Learning in Neto1-Null Mice

**Figure 10:**
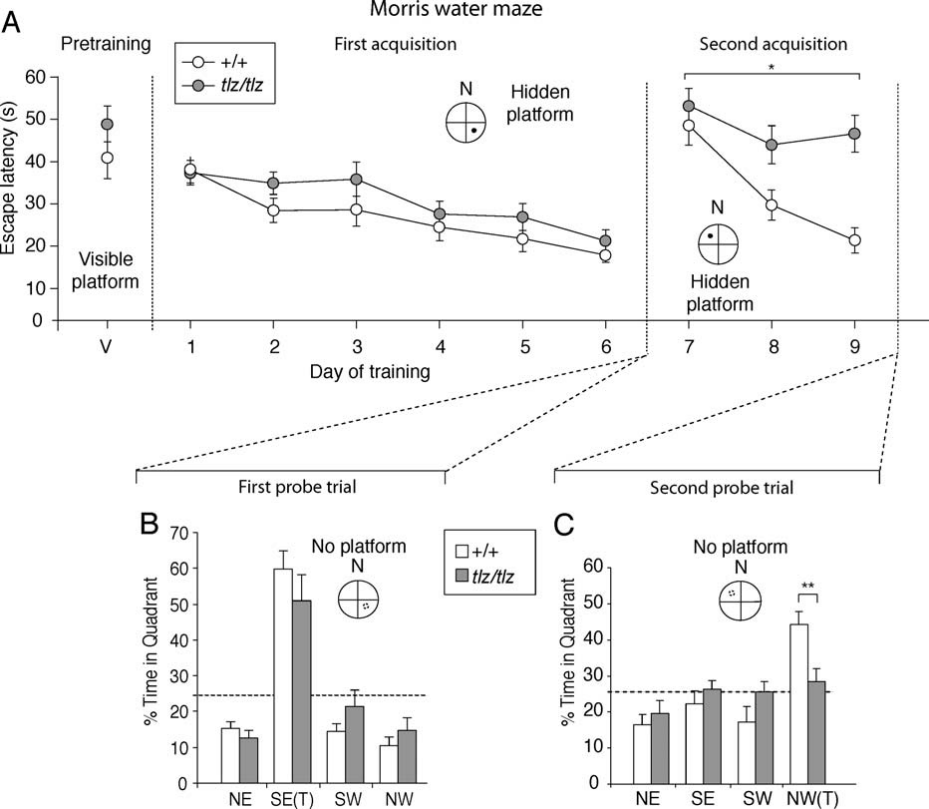
Neto1-Null Mice Have Impaired Spatial Learning and Memory (A) Latency to find the platform of wild-type (*n* = 9) and Neto1-null mice (*n* = 9) in the Morris water maze task at each day of training. During pretraining, escape latency to find a visible-cued (V) platform located in the northeast (NE) quadrant was unaffected by genotype. Similarly, in the acquisition phase (days 1–6), escape latency to find a hidden platform located in the southeast (SE) quadrant was unaffected by genotype. In the second acquisition phase (days 7–9), Neto1-null mice had longer escape latencies when the hidden platform was relocated to the northwest (NW) quadrant (effect of genotype: *F*_1,16_= 5.50, *p* < 0.05; genotype × day interaction: *F*_2,32_ = 4.17, *p <* 0.05). (B) Histogram of percent time spent in each quadrant after the first acquisition phase. T, target quadrant. (C) Histogram of percent time spent in each quadrant after the second acquisition phase. Neto1-null mice spent significantly less time in the new target quadrant (NW) than wild-type littermates (effect of genotype: *F*_1,16_ = 9.75, *p* < 0.01). Data are shown as mean ± standard error of the mean (SEM).

We reasoned that the decrease in NMDAR abundance and function in the hippocampus of Neto1-null mice might disrupt NMDAR-dependent learning and memory [3], and therefore tested wild-type and Neto1-null littermate mice in the Morris water maze task, with two acquisition phases [35]. We found no difference between wild-type and Neto1-null mice in latency to find a platform marked with a visible cue (Figure 10A, pretraining), indicating that the lack of Neto1 had no detectable adverse effects on the visual and motor functions required for this task. Moreover, there were no differences between groups in the first acquisition phase (Figure 10A, days 1–6), nor in the first probe trial (Figure 10B and Figure S7A).

In contrast, when the platform was relocated in the second acquisition phase, Neto1-null mice failed to reduce their escape latency during training and were impaired in the second probe trial as compared with the wild-type controls (Figure 10A, days 7–9). The differences could not be explained by a deficit in motor performance because swim speed, measured in every trial, was not different between the two genotypes (Figure S7B). In the second probe trial [35], wild-type mice showed a strong preference for the new target quadrant whereas Neto1-null mice showed no preference for this quadrant (Figure 10C). In addition, the mutant mice crossed the new platform location less frequently than their wild-type littermates (Figure S7C) and did not persevere in crossing the original platform location (Figure S7C). Neto1-null mice predominantly used nonspatial search strategies, such as scanning and chaining, as compared with the spatial strategies such as focal searching and direct swims [36] used by wild-type mice (Figure S7D and S7E). Altogether, the above findings establish that Neto1-null mice are impaired in hippocampal-dependent spatial learning.

**Figure 11:**
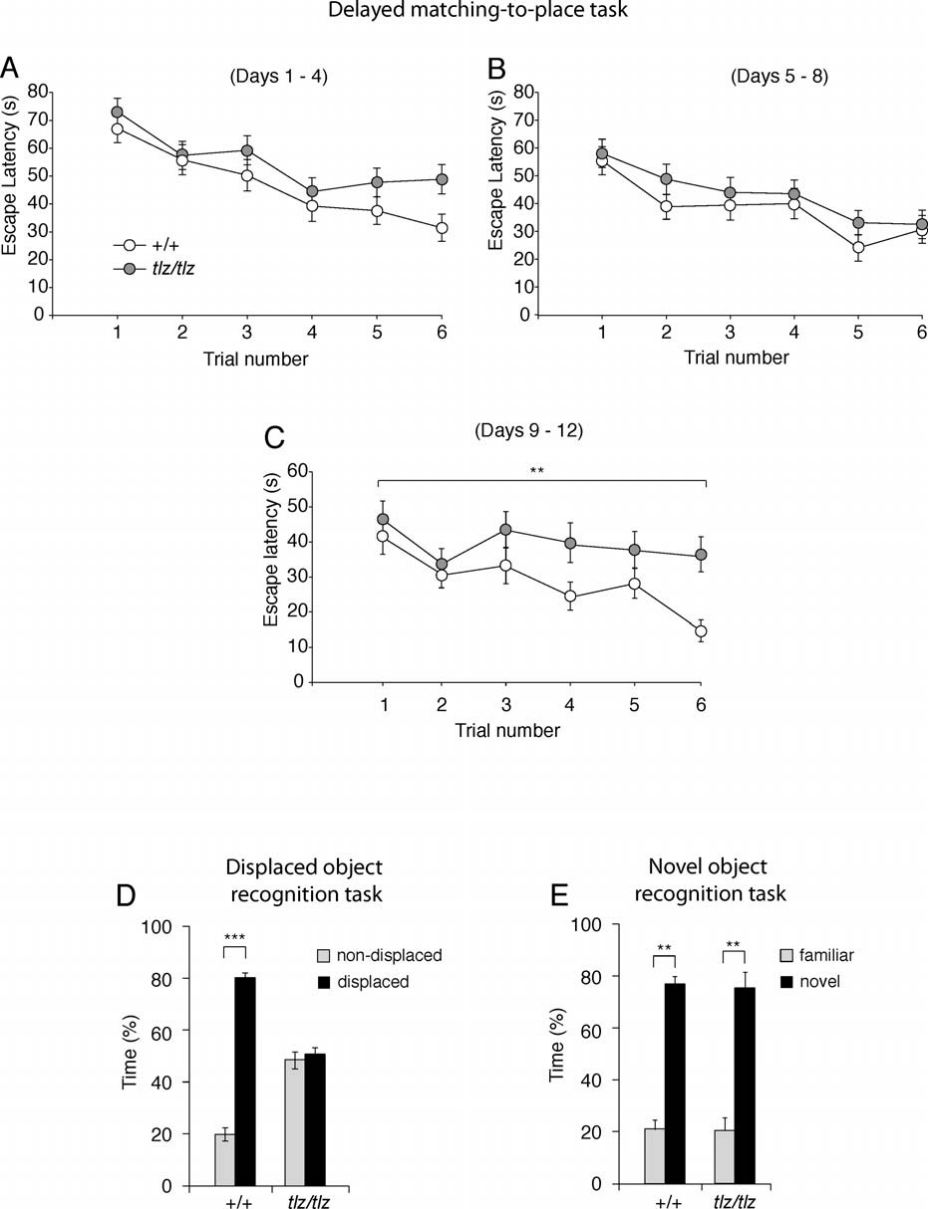
Neto1-Null Mice Are Impaired in Rapid Spatial Learning In the delayed matching-to-place (DMP) version of the Morris water maze task, wild-type and Neto1-null mice were trained each day to navigate to a new hidden platform placed in one of 12 assigned locations. (A, B) Latency to find novel platform locations during the first 8 d of training. (A) Training block 1 (days 1–4): escape latency for each trial averaged across 4 d. (B) Training block 2 (days 5–8): escape latency for each trial averaged across 4 d. (C) Latency to find novel platform locations during the last 4 d of the 12-d training period. Neto1-null mice had longer escape latencies than *Neto1^+/+^* mice (*F*_1,64_ = 9.03, *p* < 0.01) during days 9–12. For each of the six trials conducted each day, the escape latencies were averaged over multiple subjects for each genotype. **, *p* < 0.01. (D) Response to spatial novelty in wild-type (*n* = 12) and Neto1-null mice (*n* = 12). Analysis of the time spent in contact with DOs and NDOs revealed a significant effect of object rearrangement (*F*_1,22_ = 17.2, *p* < 0.001), genotype effect on time spent on DO versus NDO objects (*F*_1,22_ = 3.5, *p* < 0.05), as well as their interactions (*F*_1,22_ = 35.9, *p* < 0.001). Wild-type mice spent significantly more time examining the DO versus the NDO (*F*_1, 11_ = 78.6, *p* < 0.001), whereas Neto1-null mice spent the same time examining both the DO and the NDO (*F*_1,11_ = 1.2, *p* > 0.05). All mice had a similar latency to find the DO (wild-type: 35.3 s ± 5.7 s; Neto1-null: 31.2 s ± 12.7 s), therefore, excluding a possible influence of anxiety in response to the spatial changes and reaction to the DO. (E) Response to object replacement. Neto1-null mice were not impaired in novel object recognition. ANOVA revealed a significant effect of object novelty (*F*_1,22_ = 67.6, *p* < 0.001), no main effect of genotype on time spent on familiar object (FO) versus novel object (NO) (*F*_1,22_ = 0.04, *p* > 0.05) or their interactions (*F*_1,21_ = 0.04, *p* > 0.05). Wild-type (*F*_1,11_ = 54.0, *p* < 0.001, *n* = 12) and Neto1-null (*F*_1,11_ = 24.4, *p* < 0.001, *n* = 12) expressed marked interest to the NO versus FO. **, *p* < 0.01 in comparison with familiar object. All genotypes had the same latency to find the novel object (wild-type: 115.4 s ± 14.2 s; Neto1-null: 103.4 s ± 7.8 s). Error bars represent ± standard error of the mean (SEM).

To further characterize the hippocampal-dependent learning abnormalities in Neto1-null mice, we used two other spatial learning tests—the delayed matching-to-place version of the Morris water maze task [37] and the displaced object (DO) task [38]—and a nonspatial test, the novel object recognition task [39]. Neto1-null mice were impaired in both the delayed matching-to-place task (Figure 11A–11C and Figure S8) and the DO task (Figure 11D). In contrast, the performance of Neto1-null mice was the same as wild-type littermates in the novel-object recognition task (Figure 11E, Figure S9A, and Table S1). Taken together, our findings from the behavioural studies indicate that Neto1-null mice have broad deficiencies in spatial learning whereas the nonspatial task examined did not require Neto1.

### Rescue of tbLTP and Spatial Learning in Neto1-Null Mice by CX546

**Figure 12:**
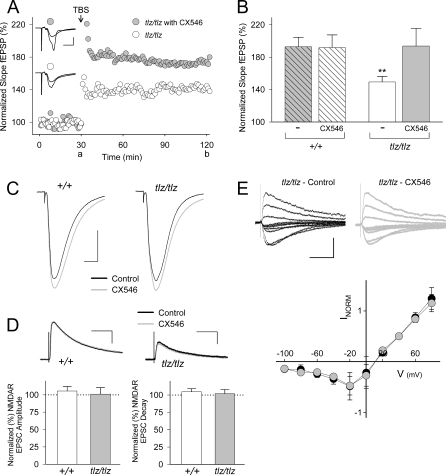
The Ampakine CX546 Restores the tbLTP Deficit in Neto1-Null Mice and Increases AMPAR, but Not NMDAR, EPSC Amplitude at Schaffer Collateral-CA1 Synapses (A) Scatter plots of normalized fEPSP slope plotted every 1 min from two individual representative Neto1-null slices without (white circle) or with (gray circle) CX546 (25 μm). When present, CX546 was applied to ACSF beginning 20–30 min before theta-burst stimulation. Theta-burst stimulation (TBS) was delivered to Schaffer collateral-CA1 synapses at the 30-min time point. The fEPSP slope was normalized with respect to the mean slope of fEPSPs recorded during the 10-min period immediately before theta-burst stimulation. Inset: average of six consecutive fEPSPs recorded at the times indicated (a or b; scale bars: 10 ms, 0.5 mV). (B) Histogram showing theta-burst stimulation-induced increase in fEPSP slope 90 min after theta-burst stimulation in slices from Neto1-null mice (*tlz/tlz*) without CX546 (-, white bar; *n* = 17 slices) and with CX546 (filled bar; *n* = 9 slices) and in *Neto1^+/+^* mice without CX546 (-, filled hatched bar; *n* = 20 slices) and with CX546 (white hatched bar; *n* = 7 slices). Results are expressed as a percentage of normalized slope fEPSP. **, *p* < 0.01 versus *tlz/tlz* with CX546 (filled bar). (C) Representative traces show AMPAR EPSCs (held at −70 mV) before (black traces) and 20 min after CX546 (25 μM; gray traces) administration in hippocampal slices from *Neto1^+/+^* (+/+) and *Neto1*-null (*tlz/tlz*) mice. Each EPSC is the average of six consecutive traces. Scale bars: 20 ms, 200 pA. (D) Representative traces show NMDAR EPSCs (+60 mV) before (black traces) and 20–30 min after CX546 (25 μM; gray traces) administration in hippocampal slices from *Neto1^+/+^* (+/+) and *Neto1*-null (*tlz/tlz*) mice. Each EPSC is the average of six consecutive traces. Scale bars: *+/+*, 100 ms, 75 pA; *tlz/tlz*, 100 ms, 35 pA. Below: Histogram of pharmacologically isolated NMDAR EPSC amplitude (left) or decay (right) from CA1 neurons in hippocampal slices from *+/+* (*n* = 8 neurons) and *tlz/tlz* (*n* = 6 neurons) mice 20–30 min after CX546 administration (25 μM; filled bars). Results are expressed as a percentage of NMDAR EPSC amplitude or decay with the amplitude or decay in *+/+* and *tlz/tlz* slices before CX546 treatment normalized to 100% (dotted line). (E) Top: superimposed NMDAR EPSC traces at *V*_m_ from −100 to +80 mV in steps of 20 mV (scale bars: 100 ms, 70 pA) from a *tlz/tlz* hippocampal CA1 neuron before (black traces) and 20 min after CX546 administration (gray traces). Bottom: Summary scatter plot shows current-voltage (I-V) relationship for pharmacologically isolated NMDARs before (black circles) and after CX546 administration (gray circles) from five *tlz/tlz* hippocampal CA1 neurons. Error bars represent ± standard error of the mean (SEM).

We considered that the deficits in LTP and learning might be restored by enhancing the residual NMDAR function in Neto1-null mice. Our strategy was to increase NMDAR-mediated currents preferentially at active synapses using the ampakine CX546. CX546 decreases the desensitization of AMPARs [15], thereby prolonging AMPAR EPSPs and secondarily increasing current through NMDARs by reducing the Mg^2+^ blockade. We found that at a concentration of 25 μM, CX546 had no effect on tbLTP in wild-type slices but restored tbLTP in Neto1-null slices to the wild-type level (Figure 12A and 12B). At this concentration, CX546 prolonged AMPAR-mediated EPSCs (Figure 12C) and this effect was similar in both wild-type and Neto1-null neurons (wild-type 160 ± 16%; Neto1-null 154 ± 21%). In contrast, CX546 (25 μM) had no effect on the amplitude, decay, or voltage-dependence of pharmacologically isolated NMDAR EPSCs (Figure 12D and 12E). CX546 (25 μM) also had no effect on paired-pulse facilitation (Figure S10A), indicating that presynaptic function was not altered by CX546. Corresponding to the prolongation of AMPAR EPSCs CX546 caused an increase in the duration of the fEPSPs (Figure S10B) and CX546-prolonged fEPSPs showed an NMDAR-component (Figure S10C). Moreover, we found that the fully rescued LTP in Neto1-null hippocampal slices was suppressed by more than 65% by Ro25–6981, at a dose that was without effect on LTP in wild-type slices (GMP, DN, RRM, MWS, unpublished data). These findings indicate that by prolonging AMPAR EPSCs, CX546 secondarily increases current through NMDARs in CA1 hippocampus in Neto1-null mice, thereby restoring tbLTP to wild-type levels.

**Figure 13:**
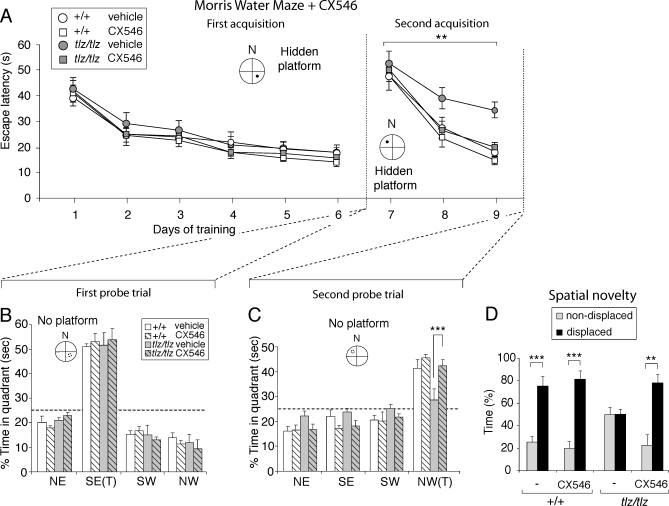
Spatial Learning Impairments in Neto1-Null Mice Are Rescued by CX546 (A) Latency to find the platform of wild-type and Neto1-null mice at each day of training in the Morris water maze task. Mice were administered either vehicle (25% cyclodextran) or 15 mg/kg CX546. During pretraining, escape latency to a visible-cued (V) platform located in the northeast (NE) quadrant was unaffected by genotype (unpublished data). In the acquisition phase (days 1–6), escape latency to find a hidden platform located in the southeast (SE) quadrant was also unaffected by genotype (*F*_1,10_ = 0.544, *p* > 0.05). In the second acquisition phase (days 7–9), Neto1-null mice treated with vehicle had longer escape latencies compared with Neto1-null mice treated with CX546, as well as wild-type mice treated with the vehicle control or CX546 when the hidden platform was relocated to the northwest (NW) quadrant (three-way ANOVA, genotype effect, *F*_1,20_ = 8.28, *p* < 0.01). In contrast, Neto1-null mice treated with CX546 had escape latencies identical to wild-type mice treated with the vehicle or CX546 (one-way ANOVA, *F*_1,15_ = 0.45; *p* = 0.7). There was no difference in escape latency between Neto1-null and wild-type mice treated with CX546 (*F*_1,10_ = 0.977, *p* > 0.05). (B) Histogram of percent time spent in each quadrant after the first acquisition phase. There were no differences between groups (one-way ANOVA, *F*_1,10_ = 0.96, *p* > 0.3). (C) Histogram of percent time spent in each quadrant after the second acquisition phase. Neto1-null mice treated with CX546 spent the same amount of time in the new target quadrant as wild-type mice treated with vehicle control or CX546. In contrast, Neto1-null mice given vehicle control did not show a preference for the new target quadrant. (D) Histogram summarizing the rescue effect of CX546 on Neto1-null mice in the spatial novelty behavioural task. Neto1-null mice treated with vehicle (*n* = 8) were impaired in spatial learning (*F*_1,14_ = 2.6, *p <* 0.05). Neto1-null mice administered CX546 (*n* = 7) were indistinguishable from spatial object recognition of wild-type controls and were able to discriminate DO versus NDO (*F*_1,12_ = 53.4, *p* < 0.001). Wild-type vehicle controls (*n* = 8) did not differ from wild-type mice given CX546 (*n* = 8) in response to spatial rearrangements (*F*_1,14_ = 3.5, *p* > 0.05). Error bars represent ± standard error of the mean (SEM).

Finally, we asked whether the strategy of using CX546 to indirectly enhance NMDAR function restores learning and memory in Neto1-null mice. In the Morris water maze task we used a dose of CX546 (15 mg/kg) that had no effect on learning in wild-type mice but that restored the escape latency and probe trial impairments in Neto1-null mice to normal (Figure 13A–13C and Figure S11). Moreover, in the DO task, Neto1-null mice treated with the same dose of CX546 spent the same amount of time investigating the DO as wild-type mice (Figure 13D). All test groups had a similar habituation profile (Figure S9B). In summary, tbLTP and spatial learning in Neto1-null mice were pharmacologically rescued by CX546, at doses that were without effect in wild-type animals.

## Discussion

We have established that Neto1 is a critical component of the NMDAR complex, and that loss of Neto1 leads to impaired hippocampal LTP and hippocampal-dependent learning and memory. We have shown that Neto1 interacts with NMDARs through the extracellular domain of their NR2 subunits, as well as intracellularly through PSD-95. Although Neto1 binds to both NR2A and NR2B, the loss of Neto1 leads to a reduction in the abundance of NR2A, but not NR2B, in the PSD fraction from hippocampus and a reduction in NR2A puncta in the CA1 region. Consistent with the reduction in NR2A protein in the PSDs of Neto1-null mice, which had no change in total NR2A abundance in whole brain, we identified a decrease in NMDAR EPSCs at Schaffer collateral-CA1 synapses, which are normally dominated by NR2A-containing receptors [40]. Blockade of NR2B-containing NMDARs in Neto1-null neurons caused a dramatic decrease in NMDAR-mediated EPSCs, indicating that the majority of NMDAR-mediated EPSCs in Neto1-null hippocampal neurons are contributed by NR2B-containing NMDARs and not NR2A-NMDARs. These findings indicate that Neto1 plays a critical role in maintaining the delivery or stability of NR2A-containing NMDARs at CA1 synapses.

The preferential effect of the loss of Neto1 on the abundance of synaptic, but not total, NR2A-containing NMDARs would not have been predicted from studies on the basis of the disruption of other NMDAR-interacting proteins. Rather than having a specific regulatory role on synaptic targeting of NMDARs like Neto1, loss of function of the other NMDAR-interacting proteins studied to date affects the overall cellular trafficking, function, or downstream signaling of NMDARs [41–43]. In its role in targeting NR2A-NMDARs to the synapse, Neto1 may be comparable to the TARPs, which control targeting of AMPARs to synapses [44,45]. Our identification of Neto1 as a critical auxiliary protein for NR2A-NMDARs raises the possibility that other proteins, perhaps other CUB domain proteins, may be required, like Neto1, to maintain non-NR2A-NMDARs at synapses. Thus, Neto1 represents a new protein that functions to specifically maintain synaptic NMDARs, a protein that has been elusive for NMDARs.

The loss of synaptic NR2A-containing receptors in the Neto1-null mice implies that the molecular events regulating the delivery or stability of NR2A-NMDARs at the synapse differ from those regulating NR2B-NMDARs. Despite the ability of Neto1 to bind to both NR2A and NR2B subunits in vitro, the differential effect of Neto1 on NR2A- versus NR2B-containing NMDARs in vivo, might be mediated by the extracellular, membrane or cytoplasmic domains of these NR2 subunits. The membrane domains of NR2A and NR2B, however, are over 95% identical and are therefore unlikely to be responsible for the differential effect of loss of Neto1. The extracellular domains of NR2A and NR2B are 54% identical, being dominated by the S1 ligand-binding region and the amino terminal domain, with the extreme N-terminal sequence being the most divergent. The cytoplasmic domains of NR2A and NR2B are the most divergent, having only 29% sequence identity. Differences in motifs within the extracellular or cytoplasmic domains may thus be responsible for the differential effect on synaptic NR2A NMDARs in the Neto1-null mice.

The functional consequences of the differences between NR2A and NR2B have been most clearly delineated for their cytoplasmic domains. For example, the endocytic motifs in the distal C termini of NR2A and NR2B, LL and YEKL, respectively, have been demonstrated to interact with clathrin adaptor complexes with different affinities [46]. After endocytosis, NR2A and NR2B sort into different intracellular pathways, with NR2B preferentially trafficking to recycling endosomes. Other studies indicate that the cytoplasmic domains of NR2A and NR2B preferentially associate with unique sets of proteins. For example, NR2B but not NR2A interacts with Ras-guanine nucleotide-releasing factor 1 (Ras-GRF1), which is critical for NMDAR-mediated activation of ERK [47]. NR2B also binds preferentially to CaMKII [48–51] allowing CaMKII to remain active after the dissociation of Ca^2+^/calmodulin. NR2 subunit-specific signalling mechanisms can therefore be dictated, in part, by the properties and context conferred by the different associated proteins. Thus, the Neto1-dependent subunit-specific regulation may reflect differences in NR2-NMDAR associated proteins.

The loss of Neto1, while having no effect on basal AMPAR-mediated synaptic transmission, suppresses LTP to a degree comparable to that observed in mice lacking NR2A [52] or its C-terminal tail [53]. In NR2A-null mutant mice, as in Neto1-null mice, LTP at Schaffer collateral-CA1 synapses is mediated by NR2B-NMDARs [54]. Moreover, the spatial memory deficit of Neto1-null mice in the Morris water maze task is comparable to that of NR2A-null mice: the initial acquisition is normal, but other tests of spatial memory are impaired including, for example, the “spontaneous spatial novelty preference test” [55]. Similarly, in mice lacking the C terminus of NR2A, the initial acquisition in the Morris water maze is normal but, like the NR2A-null, these mice also have impaired spatial working memory [55]. The deficits in the Neto1-null mice indicate that Neto1 may have specific roles in the acquisition of spatial memory. The deficit in the delayed matching-to-place indicates that Neto1 is crucial for rapid spatial learning as described by Nakazawa and colleagues [37].

Our discovery that Neto1 in vertebrates is a component of the NMDAR complex, together with the previous identification of SOL-1 [11] and LEV-10 [13] in C. elegans as CUB domain-containing proteins associated with the GLR-1 and ACh receptors, respectively, suggests that the CUB domain may be an evolutionarily conserved molecular signature of a significant subset of the proteins associated with neurotransmitter receptors. Loss of function of these three CUB domain proteins has no impact on the overall abundance of the associated receptor complexes. Rather, loss of Neto1 and LEV-10 each leads to a reduction in synaptic localization of the cognate receptors, whereas loss of SOL-1 leads to a loss of function of normally distributed GLR-1. Both Neto1 and SOL-1 interact with ionotropic subunits by an extracellular CUB domain. Binding of a soluble CUB domain of SOL-1 partially rescues the function of GLR-1 ionotropic receptors [12]. It is not yet known whether soluble Neto1 CUB domains can rescue the impaired LTP or the reduced number of NR2A-containing receptors at hippocampal excitatory synapses in Neto1-null mice. Because Neto1, SOL-1, and LEV-10 are associated with neurotransmitter receptors of different classes, our work suggests that a critical interaction with a CUB domain-containing protein may be a general characteristic of ligand-gated ion channels throughout nature.

**Figure 14:**
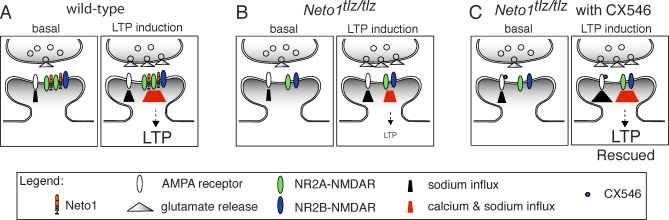
Proposed Model by Which CX546 Rescues Impaired LTP at Neto1-Null Schaffer Collateral-CA1 Synapses (A) Left: At wild-type synapses, during basal synaptic transmission, glutamate release activates AMPA receptors, causing a depolarization of the synaptic membrane. Right: During LTP induction, membrane depolarization provides a temporary relief of the magnesium ion blockade of NMDARs (primarily NR2A-NMDARs), allowing sodium and calcium ions to enter through the receptor, which triggers events leading to LTP. (B) Left: At Neto1-null Schaffer collateral-CA1 synapses, basal synaptic transmission through AMPA receptors is unperturbed. Right: During LTP induction, in the absence of Neto1, current through NMDARs (primarily mediated by NR2B-containing NMDARs) is significantly reduced, leading to a reduction in NMDA receptor signaling and impairment in LTP. (C) Left: Binding of CX546 to AMPA receptors alters the receptor desensitization kinetics and prolongs membrane depolarization, allowing more influx of sodium ions. Right: During LTP induction, at a concentration of CX546 sufficient to restore the LTP deficit in Neto1-null Schaffer collateral-CA1 synapses, prolonged membrane depolarization extends the temporary relief of the magnesium ion blockade, increasing the sodium and calcium ion influx through NMDARs to levels sufficient to restore LTP to wild-type levels.

In Neto1-null mice, the impairments in LTP and spatial learning were rescued by the ampakine CX546, administered acutely by bathing hippocampal slices in the drug prior to LTP-inducing stimulation, or by administering it systemically prior to each training session, respectively. Importantly, CX546 was used at doses that we demonstrated to have no effect on synaptic plasticity or learning in wild-type mice. This is the first report of a pharmacological rescue of an NMDAR impairment, and consequently, our results extend the principle that in vertebrates, an inherited defect in synaptic plasticity and spatial learning can be corrected in the adult [56]. We showed that CX546 prolongs AMPAR-mediated EPSCs and that the prolongation is the same in wild-type and Neto1-null mice, but that it does not affect NMDAR-mediated EPSCs or paired pulse facilitation. Consequently, the most parsimonious explanation of the CX546-mediated rescue (Figure 14) is that it indirectly facilitates NMDAR-mediated synaptic responses by prolonging AMPAR EPSCs, extending the temporary relief of the Mg^2+^ blockade and thereby increasing Ca^2+^ influx through NMDARs to the wild-type level required for full expression of the LTP signaling cascade [43,57]. A comparable strategy of modulating non-NMDARs to secondarily facilitate NMDAR currents has also been used, but with a genetic approach, in C. elegans: the disruption of foraging behaviour by mutant NMDARs was restored by a slowly desensitizing variant of the non-NMDARs [58]. Thus, we expect that a slowly desensitizing AMPAR variant would rescue LTP in the Neto1-null mice. The recovery of LTP or learning by CX546 could be explained by facilitation of either NR2A- or NR2B-NMDAR mediated responses. However, we found that the fully rescued LTP is suppressed by more than 65% in Neto1-null hippocampal slices by Ro25–6981, at a dose that is without effect on LTP in wild- type slices indicating that NR2B-NMDARs, and not only NR2A-NMDARs, are required for the rescue of LTP. Hence, the rescue of spatial learning observed in Neto1-null mice may also be dependent on NR2B-NMDARs.

In summary, in addition to the rescue of synaptic plasticity mediated by CX546, we have discovered that the CUB domain protein Neto1 is a component of the NMDAR complex and that it plays a central role in the normal function of NMDARs at hippocampal excitatory synapses. Mice lacking Neto1 have a normal abundance of NR2B-containing NMDAR receptors but a reduction of NR2A-containing receptors at hippocampal excitatory synapses. The reduction of NMDAR-mediated synaptic currents, impaired synaptic plasticity at hippocampal Schaffer collateral-CA1 synapses, and impaired spatial learning observed in the Neto1-null animals can be attributed to the decreased levels of NR2A-containing receptors at hippocampal excitatory synapses. Altogether, our findings establish that Neto1 is an important regulator of the NMDAR complex required for normal NMDAR-mediated synaptic plasticity and learning. Our results, together with the identification of the CUB domain proteins SOL-1 and LEV-10 as regulators of ionotropic receptors in nematode, suggest that a critical interaction with a CUB domain protein may be a common feature of different types of ligand-gated ion channels across species. Moreover, our studies establish the principle that inherited abnormalities of synaptic plasticity and spatial cognition due to NMDAR dysfunction can be pharmacologically corrected.

## Materials and Methods

### Neto1 cloning and gene targeting.

Human UniGene clusters were analyzed using the BLAST algorithm [59] to identify proteins with motifs suggestive of a neurodevelopmental function. One retinal UniGene cluster, Hs.60563, a partial cDNA predicting a CUB-domain ORF related to neuropilins and tolloids, which we designated *NETO1* [8], was selected for further study. Full-length mouse *Neto1* cDNAs were obtained by reverse transcription (RT)-PCR from adult mouse brain cDNA. To disrupt the *Neto1* gene by homologous recombination, we generated a targeting construct with a *tau*-*lacZ*-*loxP-pgk*-*neo-loxP* cassette cloned in-frame with the *Neto1* start codon (Figure 6A). Mouse R1 embryonic stem (ES) cells were electroporated, and positive clones were identified by Southern blotting. Two independent mouse lines were generated by blastocyst injection, and transmitting male chimeras were mated with C57BL/6J mice. A proportion of F_2_ *Neto1^tlz/tlz^* mice were observed to have infrequent myoclonic seizures commencing at the age of weaning [8]. However, no F3 *Neto1^tlz/tlz^* mice or subsequent generations exhibited seizure activity either by behavioural observation or by EEG recording. Therefore, we used only F3 and later generation *Neto1^+/tlz^* and *Neto1^tlz/tlz^* mice in the present study.

### Antibodies.

The generation of guinea pig anti-Neto1 antibodies is described elsewhere [60]. Rabbit antibodies to Neto1 were raised to the C-terminal 86 amino acids of Neto1 and prepared as described by Chow and colleagues [60], except that the antigen was further purified by electroelution from a SDS-polyacrylamide gel. Other antibodies were purchased from commercial sources. See Table S2 for details.

### Immunohistochemistry.

Immunostaining was adapted from Schneider Gasser et al. [61]. Briefly, fresh 300-μm vibratome-cut hippocampal slices, trimmed from sagittal brain slices, were fixed in 2% PFA/PBS on ice for 20 min, washed three times in PBS, and incubated “free-floating” in blocking solution (10% goat serum, 0.1% triton-X, PBS) for 1 h. Primary antibodies (see Table S2) in blocking solution were incubated with slices for 48 h under gentle agitation at 4 °C. Slices were washed three times in PBS, and incubated with appropriate secondary antibodies for 24 h under gentle agitation at 4 °C. Following incubation, slices were washed three times with PBS, transferred, and mounted on to glass slides with Immun-Mount (Thermo Scientific). Images were acquired using a Zeiss LSM 510 confocal microscope. For quantitative studies, three age-matched (2-mo-old) pairs of wild-type and Neto1-null littermates were examined. In each littermate pair, brain slices from each genotype were combined into the same well, and subsequently processed together under identical conditions, as described above. Slices were double-labeled with antibodies against Neto1 and either NR2A, NR2B, or PSD-95. All slices from the same well were mounted onto the same glass slide, and images were acquired with fixed exposure settings. Puncta from stratum radiatum in CA1 of Neto1-null and control slices were quantified using ImageJ software with identical parameters.

### Two-hybrid interaction studies.

The yeast two-hybrid system was initially used to determine whether the cytoplasmic tail of Neto1 could interact with PSD-95 and the related proteins PSD-93, SAP-102, and SAP-97. Fragments encoding the cytoplasmic region of Neto1 (amino acids 345–533) and the C-terminal mutant ΔTRV (comprising amino acids 345–530) were amplified by PCR from mouse whole brain cDNA and subcloned into the yeast vector pBD-GAL4 (Stratagene) containing the GAL4 DNA-binding domain. Full-length PSD-95, PSD-93, SAP-102, and SAP-97 cDNAs, and cDNAs encoding different parts of PSD-95 were derived from mouse brain by RT-PCR using primers designed from published DNA sequences. The cDNAs were subcloned into the yeast vector pAD-GAL4 (Stratagene). The controls used were the cytoplasmic domain of mouse neuropilin-1 [16] cloned into the pBD-GAL4 vector, and full-length NIP [24] cloned into the pAD-GAL4 vector. The yeast vectors were sequentially transformed into the Saccharomyces cerevisiae strain YRG-2 (Stratagene) and the interactions scored by growth in the absence of leucine, tryptophan, and histidine, and using a β-galactosidase filter assay.

### Mammalian expression constructs.

Full-length *Neto1* cDNA (encoding amino acids 1–533) and deletion mutants *Neto1-*Δ*TRV* (1–530), *Neto1-*Δ*20HA* (1–513), *Neto1-*Δ*20-eGFP* (1–513), *Neto1-*Δ*cyto-eGFP* (1–363), *Neto1-*Δ*cytoTM-eGFP* (1–340), *Neto1 CUB12-eGFP* (1–290), *Neto1 CUB1-eGFP* (1–162), *Nrpn1 CUB12-eGFP* (1–270) (from neuropilin-1) [16], and *CSF-1 EC-eGFP* (1–294) (from macrophage colony-stimulating factor 1 receptor) [62] were generated by PCR and subcloned into a variant of pcDNA3.1mycHisA(+) (Invitrogen) containing two copies of the influenza hemagglutinin (HA) epitope tag or the eGFP coding sequence, and sequence verified. GW1-PSD-95 (full-length human PSD-95) and pM18S-PDZ1–3 (containing PDZ domains 1, 2, and 3 of human PSD-95) have been described [63]. The NR1 construct used expresses the NR1-1a isoform, which lacks the PDZ binding motif [64].

### Cell culture and transfection.

For co-immunoprecipitation experiments, HEK293 cells were transfected using SuperFect (Qiagen). Cells transfected with NR1 and NR2 subunits of the NMDA receptors were grown in the presence of 300 μM DL-2-amino-5-phosphonovaleric acid (Sigma). 48 h after transfection, cells were washed with PBS and lysed in RIPA buffer (1 ml/100-mm plate), containing 50 mM Tris/HCl (pH 7.4), 150 mM NaCl, 1 mM EDTA, 1% Nonidet P-40, 0.1% SDS, 0.5% deoxycholate (DOC) supplemented with protease inhibitors. Lysed cells were incubated on ice for 30 min, and centrifuged at 14,000*g* for 15 min at 4 °C.

### Co-immunoprecipitation and immunoblot analysis.

Cell lysates (∼1.5 mg of protein) were incubated directly in the presence or absence of antibodies (2 μg) for periods ranging from 1 h to overnight at 4 °C on a rotating platform. Lysates were subsequently incubated with either 30 μl protein A-agarose beads (GE Healthcare) or 30 μl anti-mouse IgG beads (Sigma) for 1–5 h at 4 °C on a rotating platform. After centrifugation, beads were washed three times with RIPA buffer. Bound proteins were eluted with SDS sample buffer and subjected to SDS-PAGE and immunoblotting. For immunoprecipitation from crude synaptosomal fractions, prepared as previously described [65], 1 mg of synaptosomal protein was incubated in the presence or absence of antibodies (2 μg) or pre-immune IgGs overnight with rotation at 4 °C, and further incubated with either 30 μl protein A-agarose beads or 30 μl anti-mouse IgG beads for 3–5 h with rotation at 4 °C. After centrifugation, beads were washed three times with RIPA buffer. Bound proteins were eluted with SDS sample buffer and subjected to SDS-PAGE and immunoblotting.

### Subcellular fractionation and PSD isolation.

Subcellular fractionation of mouse brains was performed as described [66,67]. All buffers contained a cocktail of protease inhibitors (Roche). The PSD fraction was prepared from whole brains or pooled hippocampi from 2–4-mo-old mice as described previously [19], except that PSDs were extracted only once with Triton X-100. Crude synaptosomal fractions were prepared as previously described [65] from wild-type or Neto1-null brains. For protein quantification, proteins were solubilized by boiling in 1% SDS and quantitated using a detergent-compatible assay (Bio-Rad).

### Hippocampal slice biotinylation assay.

Biotinylation studies were performed as previously described with modifications [68]. Briefly, 200-μm hippocampal slices from age-matched wild-type and Neto1-null littermate mice were incubated in ACSF saturated in 95% O_2_ 5% CO_2_ at room temperature for at least 1 h. Ten slices from each genotype were incubated in 2 ml of ACSF containing 500 μg/ml biotin (Pierce), on ice, bubbled in 95% O_2_ 5% CO_2_, with gentle agitation for 1 h. Slices were washed three times in ACSF and homogenized with 1 ml of RIPA buffer with a protease inhibitor cocktail (Roche) and incubated on ice for 30 min. The homogenate was centrifuged and supernatant was collected, and quantified using the BioRad Dc protein quantification kit. 50 μg of total protein in a total volume of 300 μl was mixed with 200 μl of a 50% slurry of Neutravidin beads (Pierce) and rotated for 1 h at 4 °C. The beads (first bound fraction) were harvested by centrifugation and washed three times in RIPA buffer. The remaining supernatant was subjected to a second binding of 200 μl of 50% slurry of Neutravidin beads and rotated for 1 h at 4 °C. The beads (second bound fraction) were then centrifuged and washed three times with RIPA buffer. Samples were resolved by SDS PAGE and blotted with appropriate primary antibodies.

### Expression analysis.

A DNA fragment corresponding to the first CUB domain (CUB1) of mouse Neto1 was used to hybridize RNA blots using standard procedures. For in situ hybridizations, mouse embryos and mature tissues were fixed in PBS/4% paraformaldehyde (PFA) overnight, rinsed in PBS, and equilibrated in PBS/30% sucrose at 4 °C. In situ hybridization was adapted from an established protocol [69].

### Histological staining.

Two-month-old animals were perfused with 4% PFA in PBS and brains were sectioned and stained using hematoxylin and eosin or cresyl violet using standard methods. Brains used for Golgi staining were processed according to manufacturer*'*s directions (FD Neurotechnologies, Inc). Serial coronal and saggital brain sections were examined.

### Electrophysiological recordings.

Hippocampal slices prepared from 8–12-wk-old littermate mice were placed in a holding chamber for at least 1 h prior to recording. A single slice (300 μm) was then transferred to a recording chamber and superfused with artificial cerebrospinal fluid (ACSF) at 2 ml/min composed of 132 mM NaCl, 3 mM KCl, 1.25 mM NaH_2_PO_4_, 2 mM MgCl_2_, 11 mM D-glucose, 24 mM NaHCO_3_, and 2 mM CaCl_2_ saturated with 95% O_2_ (balance 5% CO_2_) at 28 ± 2 °C (pH 7.40; 315–325 mOsm). fEPSPs were evoked using bipolar tungsten electrodes located approximately 50 μm from the cell body layer in CA1 and were recorded using glass micropipettes filled with ACSF placed in the stratum radiatum 60–80 μm from the cell body layer. Stimulation of Schaffer collateral afferents consisted of single pulses (0.08-ms duration) delivered at 0.1 Hz. In LTP experiments, theta-burst stimulation (TBS) consisted of 15 bursts of four pulses at 100 Hz, delivered at an interstimulus interval of 200 ms. Stimulus intensity was set to 30%–35% of that which produced maximum synaptic responses. fEPSP slope was calculated as the slope of the rising phase between 10% and 60% of the peak of the response. Whole-cell EPSC recordings were done using the visualized method (Zeiss Axioskop 2FS microscope) with patch pipettes (3–5 MΩ) containing intracellular solution composed of: 132.5 mM Cs-gluconate, 17.5 mM CsCl, 10 mM HEPES, 10 mM BAPTA, 2 mM Mg-ATP, 0.3 mM GTP, 5 mM QX-314, (pH 7.25; 290 mOsm) placed in the cell body layer in the CA1. Synaptic responses were evoked with a bipolar tungsten electrode placed approximately 50 μm from the CA1 cell body layer. ACSF was supplemented with bicuculline methiodide (10 μM). AMPAR EPSCs were recorded with cells held at −70 mV. Stimulation to evoke AMPAR EPSCs consisted of single pulses (0.08-ms duration) delivered to Schaffer collateral-CA1 synapses at 0.1 Hz with increasing strength (Figure 8 and Figure S5). For each cell at each stimulus intensity tested, six consecutive EPSCs were recorded and the peak amplitudes averaged. NMDAR EPSCs were recorded from the same CA1 pyramidal neurons (Figure 8) but held at +60 mV in order to remove the NMDAR-voltage-dependent Mg^2+^ block and perfused with ACSF containing DNQX (5 μM) or CNQX (10 μM). The same stimulation protocol used to evoke AMPAR EPSCs was used to evoke NMDAR EPSCs. Current-voltage relationships for AMPAR and NMDAR EPSCs were also performed. Raw data were amplified using a MultiClamp 700A amplifier and a Digidata 1322A acquisition system sampled at 10 KHz, and analyzed with Clampfit 9.2 (Axon Instruments) and Sigmaplot 7 software. Recordings were performed with the experimenter blind to the genotype. ACSF was supplemented as indicated with Ro25–6981 (2 μM; Tocris), which was made fresh immediately before the experiment. ACSF was also supplemented as indicated with CX546 (25 μM; dissolved in H_2_O; Cortex Pharmaceuticals), which was made fresh immediately before the experiment. CX546 caused no change in the initial slope of the fEPSP but prolonged the decay phase. Data are presented as mean (±SEM). Student's *t*-test or two-way ANOVA with the Tukey test were used for statistical comparison.

Acutely dissociated hippocampal CA1 neurons were obtained from *Neto1* *^+/+^* and *Neto1* *^tlz/tlz^* mice as previously described [70]. At 20–22 °C, pyramidal CA1 neurons were voltage-clamped at −60 mV in the whole cell configuration using borosilicate micropipettes (series resistance 3–8 MΩ) filled with intracellular solution that contained (in mM): CsF 140, HEPES 10, MgCl_2_ 2, ethylene glycol-O-O'-bis(2-aminoethyl)-N,N,N′,N′-tetraacetic acid (EGTA) 10, magnesium adenosine 5”-triphosphate (MgATP) 4, buffered to a pH of 7.4 using CsOH and adjusted to an osmolality of 290–300 mOsm. The CA1 neurons were then lifted into the stream of extracellular perfusion solution containing (in mM): NaCl 140, CaCl_2_ 1.3, KCl 5.4, N-2-hydroxyethylpiperazine-N′-2-ethanesulphonic acid (HEPES) 25, glucose 33, tetrodotoxin 0.0003, and glycine 0.01, buffered to a pH of 7.4 with NaOH and adjusted to an osmolality of 320–325 mOsm. Rapid solution exchanges were accomplished by a motor-stepped fast perfusion system. NMDA-evoked current were recorded using the Multiclamp 700A amplifier with data filtered at 2 kHz, digitized using the Digidata 1322A, and acquired on-line at a sampling frequency of 10 kHz using the pCLAMP8 program. Prior to agonist exposure, a capacitance transient resulting from a 10-mV hyperpolarizing step was also recorded and used to estimate neuron size and current density in response to NMDA 1 mM. The concentration of NMDA that produced 50% of the maximal peak responses (EC_50_) and the respective Hill coefficient (*n*_H_) were determined according to the equations: 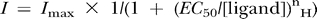 where *I*_max_ is the maximal response observed at a saturating concentration (1 mM) of NMDA (using Graphpad Prism version 4). In experiments using ifenprodil 10 μM to inhibit NR2B-containing NMDA receptors, the ifenprodil was preperfused for 2 min before its co-application with NMDA 1 mM. Data are represented as mean ±SEM.

### Behavioural testing.

For the Morris water maze task, mice tested were the 12–16-wk-old Neto1-null and wild-type F_3_ progeny of intercrossed *Neto1* ^+/tlz^ heterozygotes having a mixed genetic background averaging 50% C57BL/6J, 25% 129S1/SvImJ, and 25% 129X1/SvJ. Pink-eyed mice were excluded from behavioural testing to minimize variation in visual acuity. The water maze consisted of a 185-cm diameter cylindrical tank that contained a 15-cm circular platform and water (26 ± 1 °C) rendered opaque by the addition of white nontoxic paint. The training regime consisted of three phases: pretraining to a visible (V) platform in the northeast quadrant (NE) for 1 d (four trials; maximum duration, 90 s; inter-trial interval [ITI], 30 min); acquisition training to a hidden platform in the southeast (SE) quadrant for 6 d (day 1–6; six trials per day; maximum duration, 90 s; ITI, 40 min); second acquisition training to a hidden platform in the northwest (NW) quadrant for 3 d (day 7–9; six trials per day; maximum duration, 90 s; ITI, 30 min). Probe trials (90 s duration) were administered 18 h after the last acquisition and reversal trials, respectively.

The same cohort of mice was further trained in a delayed matching-to-place task, in which mice had to repeatedly learn a new spatial location of a hidden platform within six training trials of a daily session [71]. In this test, each mouse was given six 90 s training trials (ITI = 40 min) every day for 12 d, with the hidden platform placed in a novel location at the start of each day. The scores of each trial were averaged across the last 4 d of the 12-day training period. Behavioural data for escape latency were analysed using a two-way ANOVA. For the probe trials, statistical comparisons between genotypes for the number of crossings over the former platform location were done using one-way ANOVA with the critical α level set to 0.05 for all statistical analyses.

Swim paths of Neto1-null and wild-type mice in each trial of the second acquisition phase (Figure S7D and S7E) and delayed matching-to-place version of the Morris water maze task (Delayed Matching-to-Place [DMP] days 9–12, Figure S8A and S8B) were categorized according to their swim search strategies, as described [71,72]. Thigmotaxis: swimming along the edge of the wall or wall-hugging. Random search: randomly swimming over the entire area of the pool. Scanning: adopting a more systematic and efficient way of swimming in the central area of the pool. Chaining: memorizing a specific distance between the platform and the wall and swimming in wide circles to all possible platform locations at that distance. Focal search: restricted swimming to a specific area of the pool. Focal search signifies the beginning of spatial navigation and it could be separated into focal search in the correct target quadrant and focal search in the incorrect quadrants. The highest level of precision in spatial navigation is reached when the animal employs direct swims to the platform, independent of its release point. Swim strategies were characterized according to the predominant swim strategy used during the entire length of each trial and overall swim strategies were presented as the percentage of time spent on the strategy of choice. The experimenter classifying the swim search strategies was blind to the genotype or trial sequence within the experiment. The chaining parameter in the Wintrack computer software [36] was used to statistically verify qualitative swim search strategies of Neto1-null and wild-type mice during the second acquisition phase of the Morris water maze task and days 9–12 of the DMP task. The chaining score comparisons between genotypes were analyzed using ANOVA.

The modified open field procedure was performed as described [73], with slight modifications, using a second cohort of Neto1-null and wild-type littermate mice. The open field apparatus consisted of a cubical box (41 × 41 × 33 cm) made of clear Perspex (Ugo Basile) that was connected to horizontal and vertical infrared sensors. All behavioural events were video recorded and analyzed using Observer 5.0 software (Noldus Information Technology). The test consisted of four sessions with intertrial intervals of 2 min during which mice were returned to their home cage. During the open field session, each mouse was placed into the center of the empty, brightly lit open field for 5 min and the baseline level of locomotion (horizontal and vertical activity) and other behavioural parameters were recorded. The behavioural parameters were latency to escape the center; time of freezing (remaining in one place with only slight movement of the head); time of self-grooming; number of risk assessments (behaviour involving the mouse stretching its body from the corners/wall towards the center). Exploratory activity and walking were recorded separately for the central and peripheral field of the open arena, and the ratio between duration of central and peripheral activity was calculated.

During the habituation session, four different plastic objects were presented in the open field: cube (5 × 5 × 5 cm); hollow cylinder (6 cm height and 4 cm diameter); solid cylinder (3 cm height × 6 cm diameter); and prism (3.5 × 4.5 × 6 cm). Exploration of the four different plastic objects in the open field were measured every 5 min for 15 min under dim lighting (habituation profile). In the spatial object recognition session, the four objects, initially placed in a square arrangement, were reconfigured into a polygon-shaped pattern by moving two DOs. The remaining two objects were left at the same location (nondisplaced objects [NDOs]). Times of exploration of the DO and NDO were recorded for 5 min and expressed as a percentage of the total time of objects investigated. In the novel object recognition session, one of the familiar NDOs was replaced with a new object (NO) at the same location and the two familiar DOs were removed. The time examining a NO or a familiar object (FO) was recorded for 5 min and was expressed as a percentage of the total time of objects investigated. Data were analyzed with ANOVA with genotype as a between-subjects factor, and object rearrangement or object replacement as a repeated measures factor. The Tukey test was used for post hoc comparisons when ANOVA yielded statistically significant main effects or interactions.

To examine the effects of CX546 in spatial learning, new cohorts of Neto1-null and wild-type littermate mice were used for the water maze and displaced-object tasks. In the water maze task, a single daily intraperitoneal injection of CX546 (15 mg/kg, dissolved in 25% cylcodextran) or vehicle (25% cyclodextran) was administered 30 min prior to training. No injection was given on probe trial days. For the displaced-object task, a single intraperitoneal injection of CX546 (15 mg/kg) or vehicle was administered 30 min prior to displaced-object recognition testing.

All animal procedures were conducted in accordance with the requirements of the Province of Ontario Animals for Research Act, 1971 and the Canadian Council on Animal Care (CCAC 1984, 1995).

### Accession numbers.

GenBank (http://www.ncbi.nlm.nih.gov/Genbank) accession numbers discussed in this paper are: PSD-95 (D50621); PSD-93 (AF388675); SAP-102 (D87117); and SAP-97 (NM_007862).

## Supporting Information

Figure S1 *Neto1* Is Expressed throughout the Nervous System Top: Adult mouse multitissue RNA blot hybridized with a *Neto1* cDNA probe. The size of the three predominant *Neto1* bands is indicated on the left. RNA blotting with different *Neto1* cDNA probes and DNA sequence analysis indicate that the multiple bands observed are likely due to alternative splicing of the 3′UTR and use of different polyadenylation signals (unpublished data). Bottom: ethidium bromide staining of gel prior to blotting.
(661 KB PDF)

Figure S2 Neto1 Binding to PDZ Domains of PSD-95 Requires the C-Terminal PDZ Tripeptide In the yeast two-hybrid system, the strength of the interaction between a Neto1 cytoplasmic domain (Neto1-cd) construct, or a Neto1 mutants constructs lacking the last three amino acids (Neto1-cdΔTRV), five amino acids (Neto1-cdΔ5), ten amino acids (Neto1-cdΔ10), 20 amino acids (Neto1-cdΔ20), and PSD-95 deletion constructs is shown: +++, strong interaction; ++, moderate interaction; +, weak interaction; -, no detectable interaction; nd, no data.
(469 KB PDF)

Figure S3 Loss of Neto1 Does Not Alter the Number of NR2B or PSD-95 Puncta in CA1 Stratum Radiatum (A, B) Confocal micrographs of immunostained hippocampal slices from the CA1 region. Antibodies used are indicated in each box. Scale bar, 10 μm. Pyr, pyramidal cell layer; SR; stratum radiatum.
(2.83 MB PDF)

Figure S4 Loss of Neto1 Does Not Alter Surface Expression or Function of NMDARs (A) Immunoblots of biotinylated hippocampal surface proteins. Lanes 1, 2: biotinylated proteins after initial binding to avidin beads. Lanes 3, 4: biotinylated proteins recovered from supernatant after a subsequent binding to fresh avidin beads (i.e., remaining biotinylated protein in supernatant not captured after initial binding to avidin beads). The lack of biotinylated protein detected in lanes 3 and 4 indicates that the binding capacity of avidin beads used in lanes 1 and 2 was not exceeded. Blots shown are representative of three separate experiments.
(B) Histogram of peak current densities evoked by NMDA 1 mM from wild-type (white bar) and *Neto1*-null (gray bar) neurons. There was no significant difference between the mean NMDA peak current density calculated in *Neto1*-null neurons (244.5 ± 34.7 pA/pF, *n =* 22) compared with wild-type neurons (318.5 ± 49.4 pA/pF, *n =* 18) (unpaired *t*-test, *p* = 0.23).
(C) Histogram depicting the mean fraction of NMDA 1 mM peak current inhibited by ifenprodil 10 μM in wild-type (white bar) and *Neto1*-null (gray bar) neurons. There was no significant difference between mean fraction of NMDA current inhibited by ifenprodil in the wild-type (0.38 ± 0.05, *n* = 8) and *Neto1*-null neurons (0.36 ± 0.04, *n* = 10) (unpaired *t*-test, *p* = 0.808). Error bars represent ± standard error of the mean (SEM).
(849 KB PDF)

Figure S5 Normal Basal AMPAR EPSC Amplitude But Reduced Basal NMDAR EPSC Amplitude at Schaffer Collateral-CA1 Synapses of Neto1-Null Mice Top histogram shows peak amplitude of AMPAR EPSCs from *Neto1^+/+^* (open bars) or Neto1-null (filled bars) mice. Bottom histogram shows peak amplitude of NMDAR-mediated EPSC synaptic responses recorded from CA1 pyramidal neurons from +/+ (*n* = 20 neurons) or *tlz/tlz* (*n* = 13 neurons) mice (*, *p* < 0.05; **, *p* < 0.01, versus +/+). Strength of Schaffer collateral stimulation is indicated on the horizontal axis.
(186 KB PDF)

Figure S6 Increased Sensitivity of NMDAR EPSCs to Ro25–6981 at Schaffer Collateral-CA1 Synapses in Neto1-Null Mice Example representative NMDAR EPSCs from *Neto1^+/+^* (*+/+* NMDAR EPSCs) and Neto1-null (*tlz/tlz* NMDAR EPSCs) neurons before (I_NMDAR_) and 40 min after (I_Ro-resistant_) Ro25–6981 (2 μM) administration (scale bars: 150 ms, 50 pA). Each EPSC is the average of six consecutive traces. I_Ro-resistant_ EPSCs are also shown scaled (I_Ro-resistant_ normalized, gray) to the peak of the NMDAR EPSC before Ro25–6981 administration (I_NMDAR_, black).
(83 KB PDF)

Figure S7 Neto1-Null Mice Have Impaired Spatial Learning in the Morris Water Maze Task (A) Number of crossings over the hidden platform location (SE) after the first acquisition phase. *Neto1^+/+^* and Neto1-null mice crossed the target platform location (T) with equal frequency.
(B) Average swim speed was not different between *Neto1^+/+^* (+/+) and Neto1-null (*tlz/tlz*) mice.
(C) Number of crossings over the hidden platform location (NW) after the second acquisition phase. Neto1-null mice crossed the new target platform location (NW) less frequently than wild-type mice (one-way ANOVA *F*_1,16_ = 10.36, *p* < 0.01). Error bars shown are ± standard error of the mean (SEM).
(D, E) Swim search strategies used by *Neto1^+/+^* (*n* = 9) and Neto1-null mice (*n* = 9). (D) During the second acquisition phase in the Morris water maze, *Neto1* ^+/+^ mice predominantly used spatial strategies (focal searching and direct swims) to navigate to the relocated hidden platform during the last 2 d of the second acquisition phase (days 8 and 9 in Figure 10A). (E) In contrast, Neto1-null mice persistently used less efficient nonspatial swim strategies (chaining and scanning) throughout the second acquisition period (genotype effect on chaining, *F*_1,48_= 6.22, *p* < 0.05). Data in (D) and (E) represent the breakdown of each search strategy employed by each genotype during the second acquisition period (days 7–9 in Figure 10A).
(66 KB PDF)

Figure S8 Neto1-Null Mice Are Impaired in the Delayed Matching-to-Place Version of the Morris Water Maze Task (A) *Neto1* ^+/+^ mice used nonspatial swimming strategies (chaining and scanning) in the initial trials and then switched to spatial strategies (focal searching and direct swims) in later trials to locate the hidden platform.
(B) Neto1-null mice, however, predominantly used only nonspatial strategies (chaining and scanning), throughout the task, to navigate to the hidden platform. Data represent the breakdown of each search strategy employed during days 9–12 of the delayed matching-to-place (DMP) task.
(64 KB PDF)

Figure S9 Habituation Profile of Object Exploration in the DO Recognition Task (A) *Neto1* ^+/+^ (*n* = 12) and Neto1-null (*n* = 12) mice both showed similar exploration of objects during the habituation session. As expected, all mice spent more time exploring the object during the first 5 min, after which their exploration of objects declined. ANOVA did not find a main effect of genotype on time spent in contact with objects during the habituation period (*F*_1,22_ = 0.13, *p* > 0.05). Analysis of repeated measures revealed the main effect of habituation of time spent in contact with objects across the testing intervals (*F*_2,44_ = 123.9, *p* < 0.001). Both *Neto1* ^+/+^ and Neto1-null mice significantly decreased the time of investigation of objects (all *p* values <0.001 in comparison with first 5 min of exploration for *Neto1* ^+/+^ and Neto1-null mice).
(B) Habituation profile of object exploration in the DO recognition task of *Neto1* ^+/+^ mice administered vehicle (*n* = 7), *Neto1* ^+/+^ mice administered 15 mg/kg CX546 (*n* = 9), Neto1-null mice administered vehicle (*n* = 8), and Neto1-null mice administered 15 mg/kg CX546 (*n* = 7). ANOVA did not find a main effect of genotype or drug treatment on time spent in contact with objects during the habituation period (both *p* values >0.05). Analysis of repeated measures revealed the main effect of habituation of time spent in contact with objects across the testing intervals (*F*_2,44_ = 123.9, *p* < 0.001). Vehicle- and CX546-treated *Neto1* ^+/+^ and vehicle-treated Neto1-null mice significantly decreased the time of investigation of objects (all *p* values <0.001 in comparison with first 5 min of exploration of vehicle-treated mice within each genotype). Neto1-null mice administered CX546 significantly decreased their exploratory activity after 10 min of habituation (*p* < 0.001 in comparison with first 5 min of vehicle-treated Neto1-null mice). All groups spent more time exploring the object during the first 5 min, after which, their exploration of objects declined. Error bars represent ± standard error of the mean (SEM).
(46 KB PDF)

Figure S10 CX546 Is without Effect on Paired-Pulse Facilitation of fEPSPs at Neto1-Null Schaffer Collateral-CA1 Synapses but Enhances fEPSPs and Induces an NMDAR-Mediated Component of the fEPSPs (A) Paired-pulse facilitation of fEPSPs in Neto1-null slices treated with (*tlz/tlz* with CX546; gray circles; *n* = 9) and without (*tlz/tlz*; black circles; *n* = 5) CX546 (25 μM). Interstimulus interval is indicated on the horizontal axis. P1, fEPSP slope first response; P2, fEPSP slope second response.
(B) Representative traces show fEPSPs before (black trace) and 20–30 min after CX546 (25 μM; gray trace) administration in a hippocampal slice from a *Neto1*-null (*tlz/tlz*) mouse. Each fEPSP is the average of six consecutive traces. Scale bars: 5 ms, 0.2 mV.
(C) Each trace shows the average difference plots before (*n* = 6 consecutive control fEPSPs) minus during D-APV (80 μM; *n* = 6 consecutive fEPSPs) from a single hippocampal slice from a Neto1-null mouse. Left trace was before administering CX546 and the right trace was during bath application of CX546 (25 μM). D-APV was washed out for 40 min before administering CX546. Scale bars: 10 ms, 0.1 mV.
(150 KB PDF)

Figure S11 Impaired Spatial Learning in the Morris Water Maze Task Is Rescued by the Ampakine CX546 (A) *Neto1^+/+^* and Neto1-null mice crossed the platform location with equal frequency in the first acquisition regardless of whether they were administered vehicle or CX546 (one-way ANOVA, *F*_3,20_ = 1.78, *p =* 0.2).
(B) Average swim speed was not different between *Neto1^+/+^* and Neto1-null mice administered vehicle or 15 mg/kg CX546. Post hoc analysis did not indicate a difference in swim speed across groups (*p* > 0.6).
(C) Neto1-null mice administered CX546 crossed the platform location in the second acquisition phase with equal frequency as compared to *Neto1^+/+^* mice (*p* > 0.4). Neto1-null mice administered vehicle crossed the hidden platform location significantly fewer times compared with *Neto1^+/+^* mice given vehicle (*F*_1,10_ = 0.62; *p* < 0.05). Error bars represent ± standard error of the mean (SEM).
(54 KB PDF)

Table S1 Performance of *Neto1^+/+^* (*n =* 12) and Neto1-Null (*n =* 12) Mice in the Open Field Test (13 KB PDF)

Table S2 List of Antibodies Used in Study (47 KB PDF)

## Abbreviations

AMPAR: α-amino-3-hydroxy-5-methyl-4-isoxazolepropionic acid receptor
DO: displaced object
EPSC: excitatory postsynaptic current
fEPSP: field excitatory postsynaptic potential
LTP: long-term potentiation
NDO: nondisplaced object
NMDAR: N-methyl-D-aspartate receptor
Neto1: neuropilin tolloid-like 1
PSD: postsynaptic density
tbLTP: theta-burst pattern long-term potentiation
