# Supplementary figures and images for: Neto1 Is a Novel CUB-Domain NMDA Receptor–Interacting Protein Required for Synaptic Plasticity and Learning
Source: PLoS Biol. 2009 Feb 24;7(2):e1000041. doi: 10.1371/journal.pbio.1000041 (PMC2652390; doi:10.1371/journal.pbio.1000041)

Figure S1

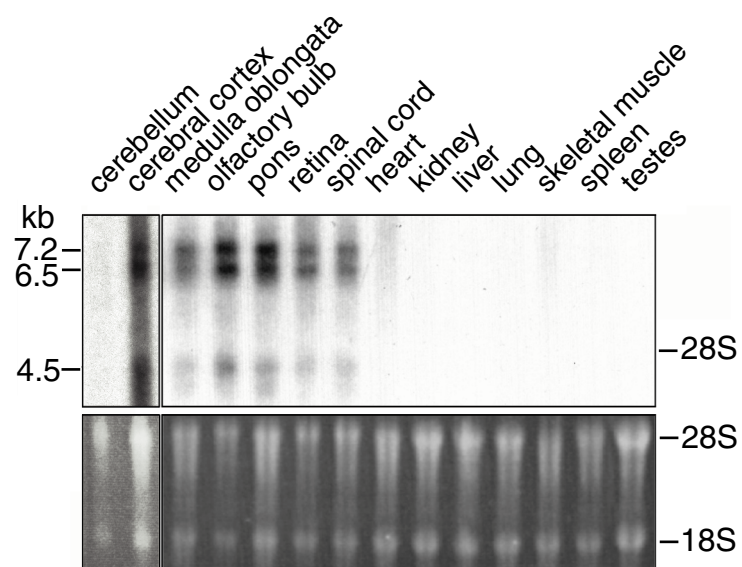

Supplement: Figure S1 — Top: Adult mouse multitissue RNA blot hybridized with a Neto1 cDNA probe. The size of the three predominant Neto1 bands is indicated on the left. RNA blotting with different Neto1 cDNA probes and DNA sequence analysis indicate that the multiple bands observed are likely due to alternative splicing of the 3′UTR and use of different polyadenylation signals (unpublished data). Bottom: ethidium bromide staining of gel prior to blotting. (661 KB PDF) [file pbio.1000041.sg001.pdf]

Figure S2

Yeast two-hybrid

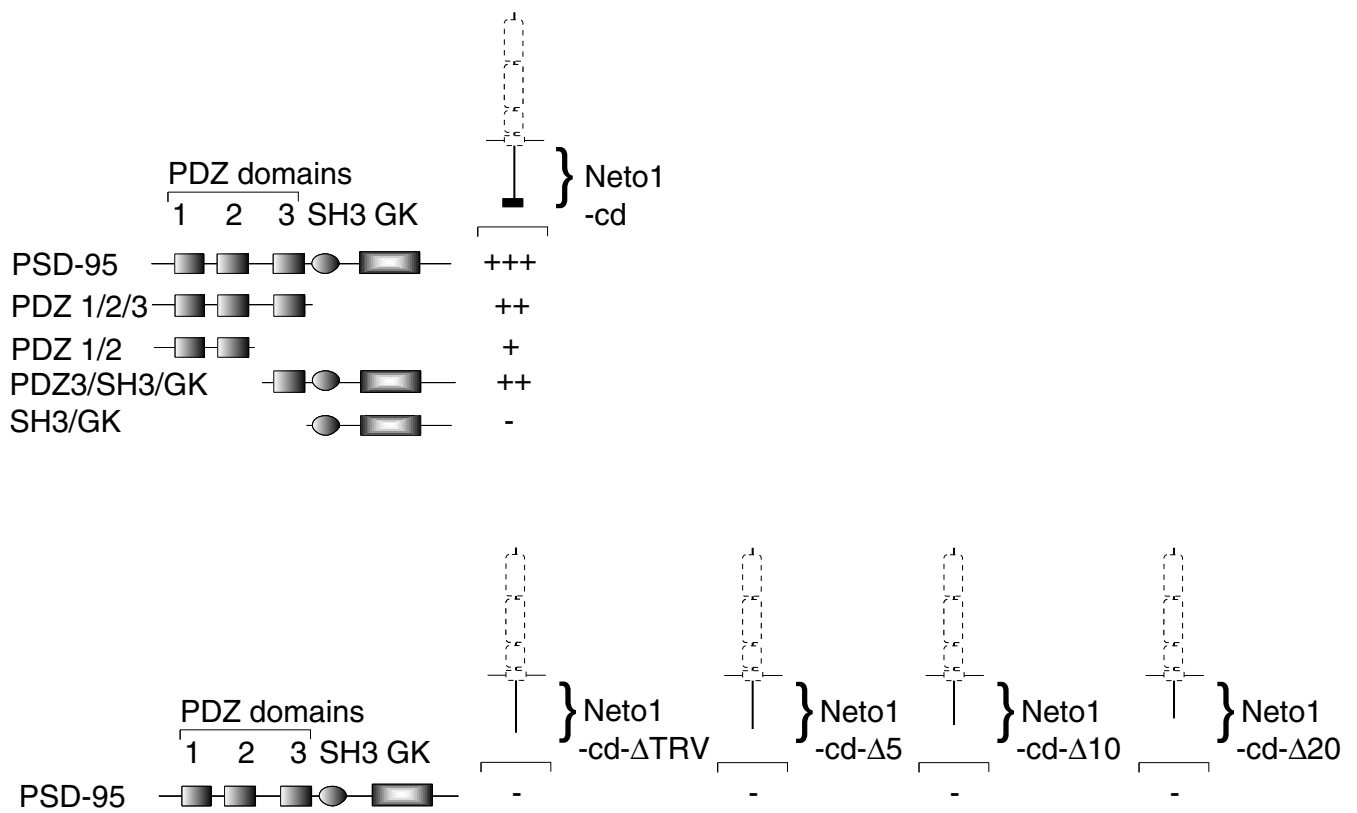

Supplement: Figure S2 — In the yeast two-hybrid system, the strength of the interaction between a Neto1 cytoplasmic domain (Neto1-cd) construct, or a Neto1 mutants constructs lacking the last three amino acids (Neto1-cdΔTRV), five amino acids (Neto1-cdΔ5), ten amino acids (Neto1-cdΔ10), 20 amino acids (Neto1-cdΔ20), and PSD-95 deletion constructs is shown: +++, strong interaction; ++, moderate interaction; +, weak interaction; -, no detectable interaction; nd, no data. (469 KB PDF) [file pbio.1000041.sg002.pdf]

Figure S3

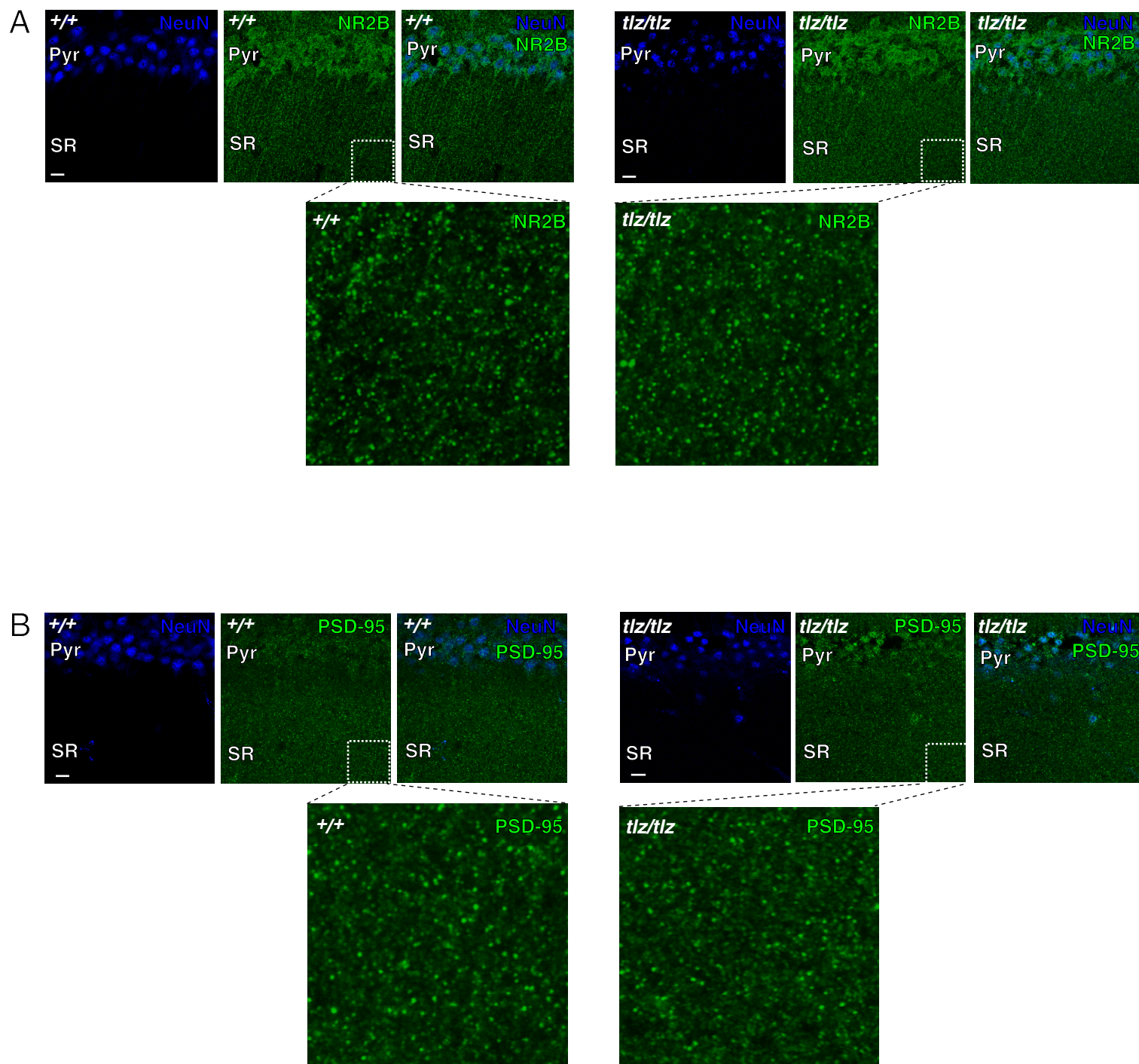

Supplement: Figure S3 — (A, B) Confocal micrographs of immunostained hippocampal slices from the CA1 region. Antibodies used are indicated in each box. Scale bar, 10 μm. Pyr, pyramidal cell layer; SR; stratum radiatum. (2.83 MB PDF) [file pbio.1000041.sg003.pdf]

Figure S4

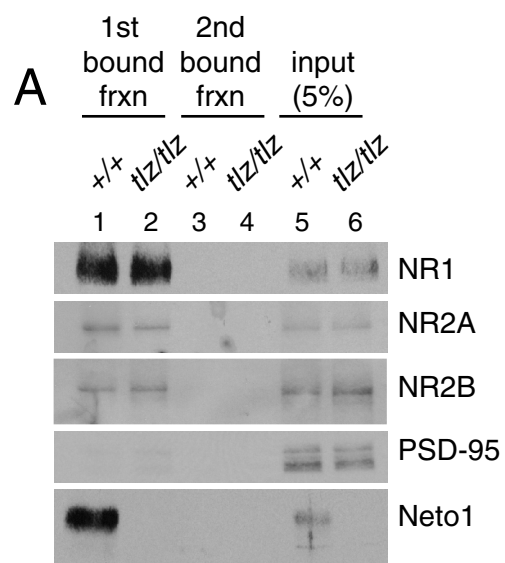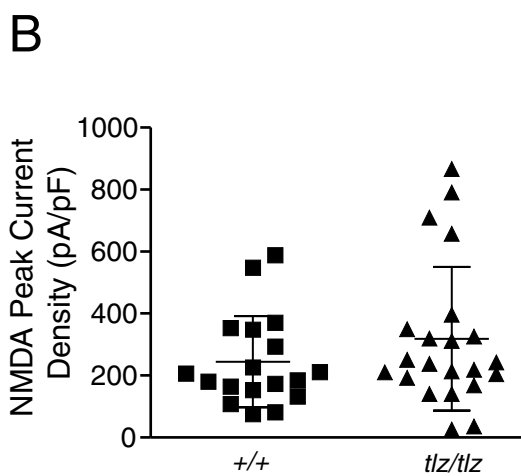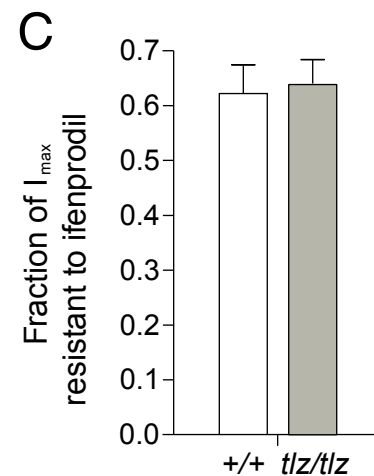

Supplement: Figure S4 — (A) Immunoblots of biotinylated hippocampal surface proteins. Lanes 1, 2: biotinylated proteins after initial binding to avidin beads. Lanes 3, 4: biotinylated proteins recovered from supernatant after a subsequent binding to fresh avidin beads (i.e., remaining biotinylated protein in supernatant not captured after initial binding to avidin beads). The lack of biotinylated protein detected in lanes 3 and 4 indicates that the binding capacity of avidin beads used in lanes 1 and 2 was not exceeded. Blots shown are representative of three separate experiments. (B) Histogram of peak current densities evoked by NMDA 1 mM from wild-type (white bar) and Neto1-null (gray bar) neurons. There was no significant difference between the mean NMDA peak current density calculated in Neto1-null neurons (244.5 ± 34.7 pA/pF, n = 22) compared with wild-type neurons (318.5 ± 49.4 pA/pF, n = 18) (unpaired t-test, p = 0.23). (C) Histogram depicting the mean fraction of NMDA 1 mM peak current inhibited by ifenprodil 10 μM in wild-type (white bar) and Neto1-null (gray bar) neurons. There was no significant difference between mean fraction of NMDA current inhibited by ifenprodil in the wild-type (0.38 ± 0.05, n = 8) and Neto1-null neurons (0.36 ± 0.04, n = 10) (unpaired t-test, p = 0.808). Error bars represent ± standard error of the mean (SEM). (849 KB PDF) [file pbio.1000041.sg004.pdf]

Figure S5

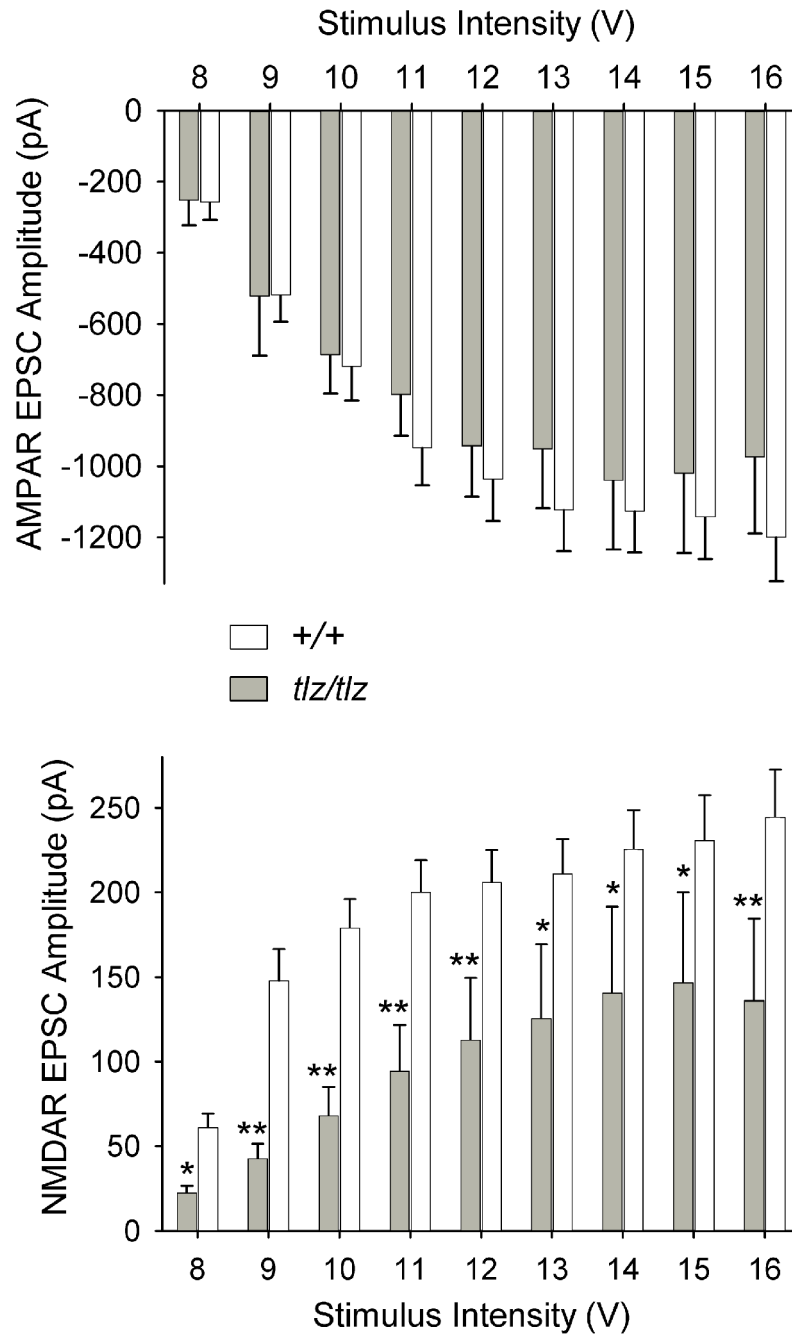

Supplement: Figure S5 — Top histogram shows peak amplitude of AMPAR EPSCs from Neto1+/+ (open bars) or Neto1-null (filled bars) mice. Bottom histogram shows peak amplitude of NMDAR-mediated EPSC synaptic responses recorded from CA1 pyramidal neurons from +/+ (n = 20 neurons) or tlz/tlz (n = 13 neurons) mice (*, p < 0.05; **, p < 0.01, versus +/+). Strength of Schaffer collateral stimulation is indicated on the horizontal axis. (186 KB PDF) [file pbio.1000041.sg005.pdf]

**+/+ NMDAR EPSCs**

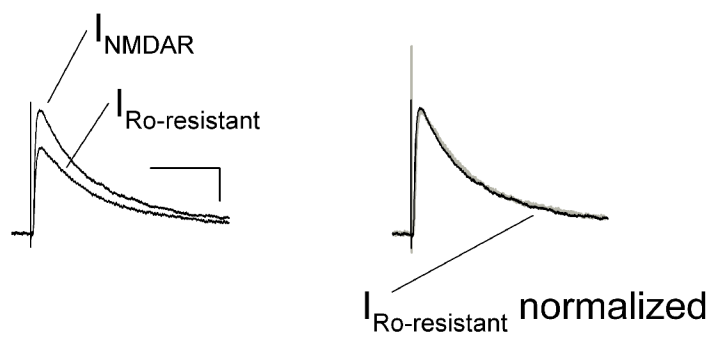

***tlz/tlz* NMDAR EPSCs**

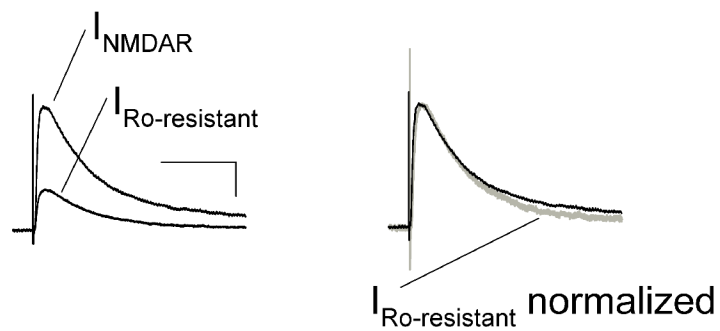

Supplement: Figure S6 — Example representative NMDAR EPSCs from Neto1+/+ (+/+ NMDAR EPSCs) and Neto1-null (tlz/tlz NMDAR EPSCs) neurons before (INMDAR) and 40 min after (IRo-resistant) Ro25–6981 (2 μM) administration (scale bars: 150 ms, 50 pA). Each EPSC is the average of six consecutive traces. IRo-resistant EPSCs are also shown scaled (IRo-resistant normalized, gray) to the peak of the NMDAR EPSC before Ro25–6981 administration (INMDAR, black). (83 KB PDF) [file pbio.1000041.sg006.pdf]

Figure S7

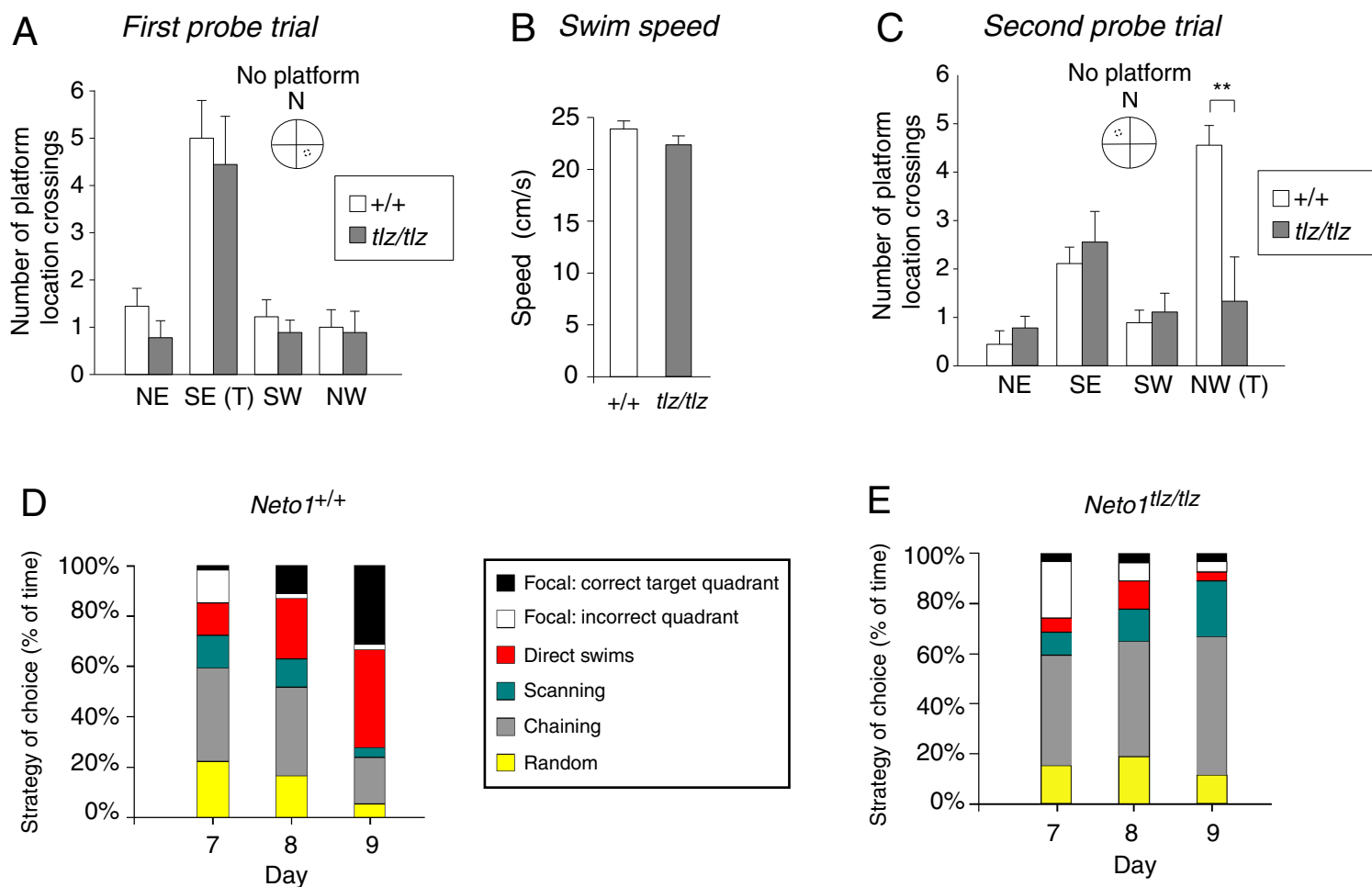

Supplement: Figure S7 — (A) Number of crossings over the hidden platform location (SE) after the first acquisition phase. Neto1+/+ and Neto1-null mice crossed the target platform location (T) with equal frequency. (B) Average swim speed was not different between Neto1+/+ (+/+) and Neto1-null (tlz/tlz) mice. (C) Number of crossings over the hidden platform location (NW) after the second acquisition phase. Neto1-null mice crossed the new target platform location (NW) less frequently than wild-type mice (one-way ANOVA F 1,16 = 10.36, p < 0.01). Error bars shown are ± standard error of the mean (SEM). (D, E) Swim search strategies used by Neto1+/+ (n = 9) and Neto1-null mice (n = 9). (D) During the second acquisition phase in the Morris water maze, Neto1 +/+ mice predominantly used spatial strategies (focal searching and direct swims) to navigate to the relocated hidden platform during the last 2 d of the second acquisition phase (days 8 and 9 in Figure 10A). (E) In contrast, Neto1-null mice persistently used less efficient nonspatial swim strategies (chaining and scanning) throughout the second acquisition period (genotype effect on chaining, F 1,48= 6.22, p < 0.05). Data in (D) and (E) represent the breakdown of each search strategy employed by each genotype during the second acquisition period (days 7–9 in Figure 10A). (66 KB PDF) [file pbio.1000041.sg007.pdf]

Figure S8

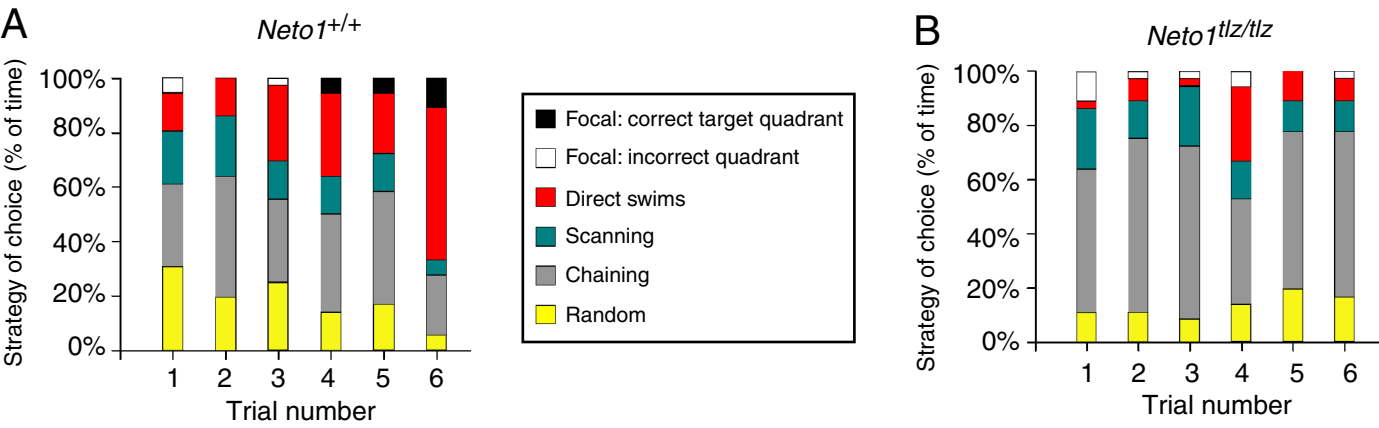

Supplement: Figure S8 — (A) Neto1 +/+ mice used nonspatial swimming strategies (chaining and scanning) in the initial trials and then switched to spatial strategies (focal searching and direct swims) in later trials to locate the hidden platform. (B) Neto1-null mice, however, predominantly used only nonspatial strategies (chaining and scanning), throughout the task, to navigate to the hidden platform. Data represent the breakdown of each search strategy employed during days 9–12 of the delayed matching-to-place (DMP) task. (64 KB PDF) [file pbio.1000041.sg008.pdf]

Figure S9

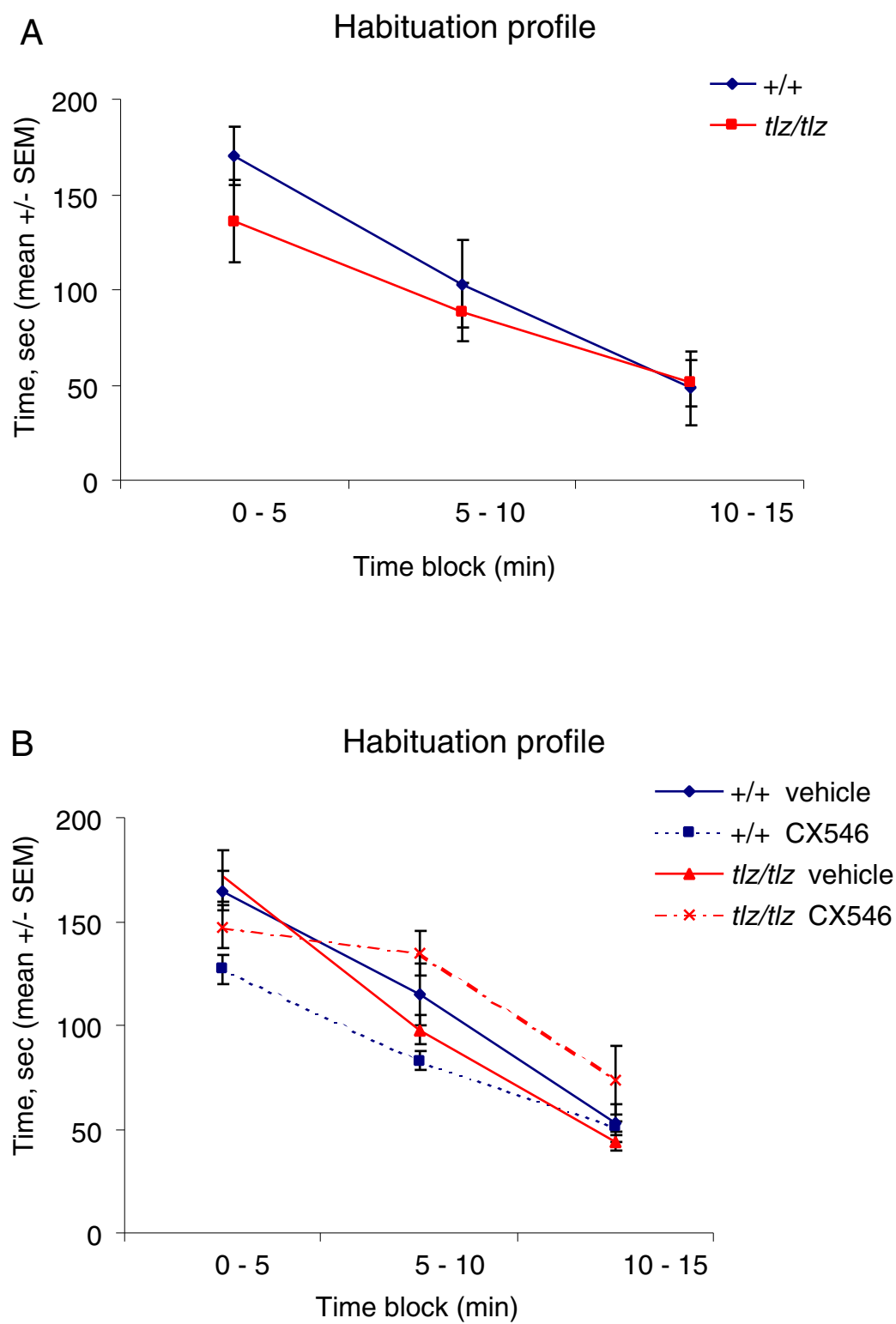

Supplement: Figure S9 — (A) Neto1 +/+ (n = 12) and Neto1-null (n = 12) mice both showed similar exploration of objects during the habituation session. As expected, all mice spent more time exploring the object during the first 5 min, after which their exploration of objects declined. ANOVA did not find a main effect of genotype on time spent in contact with objects during the habituation period (F 1,22 = 0.13, p > 0.05). Analysis of repeated measures revealed the main effect of habituation of time spent in contact with objects across the testing intervals (F 2,44 = 123.9, p < 0.001). Both Neto1 +/+ and Neto1-null mice significantly decreased the time of investigation of objects (all p values <0.001 in comparison with first 5 min of exploration for Neto1 +/+ and Neto1-null mice). (B) Habituation profile of object exploration in the DO recognition task of Neto1 +/+ mice administered vehicle (n = 7), Neto1 +/+ mice administered 15 mg/kg CX546 (n = 9), Neto1-null mice administered vehicle (n = 8), and Neto1-null mice administered 15 mg/kg CX546 (n = 7). ANOVA did not find a main effect of genotype or drug treatment on time spent in contact with objects during the habituation period (both p values >0.05). Analysis of repeated measures revealed the main effect of habituation of time spent in contact with objects across the testing intervals (F 2,44 = 123.9, p < 0.001). Vehicle- and CX546-treated Neto1 +/+ and vehicle-treated Neto1-null mice significantly decreased the time of investigation of objects (all p values <0.001 in comparison with first 5 min of exploration of vehicle-treated mice within each genotype). Neto1-null mice administered CX546 significantly decreased their exploratory activity after 10 min of habituation (p < 0.001 in comparison with first 5 min of vehicle-treated Neto1-null mice). All groups spent more time exploring the object during the first 5 min, after which, their exploration of objects declined. Error bars represent ± standard error of the mean (SEM). (46 KB PDF) [file pbio.1000041.sg009.pdf]

Figure S10

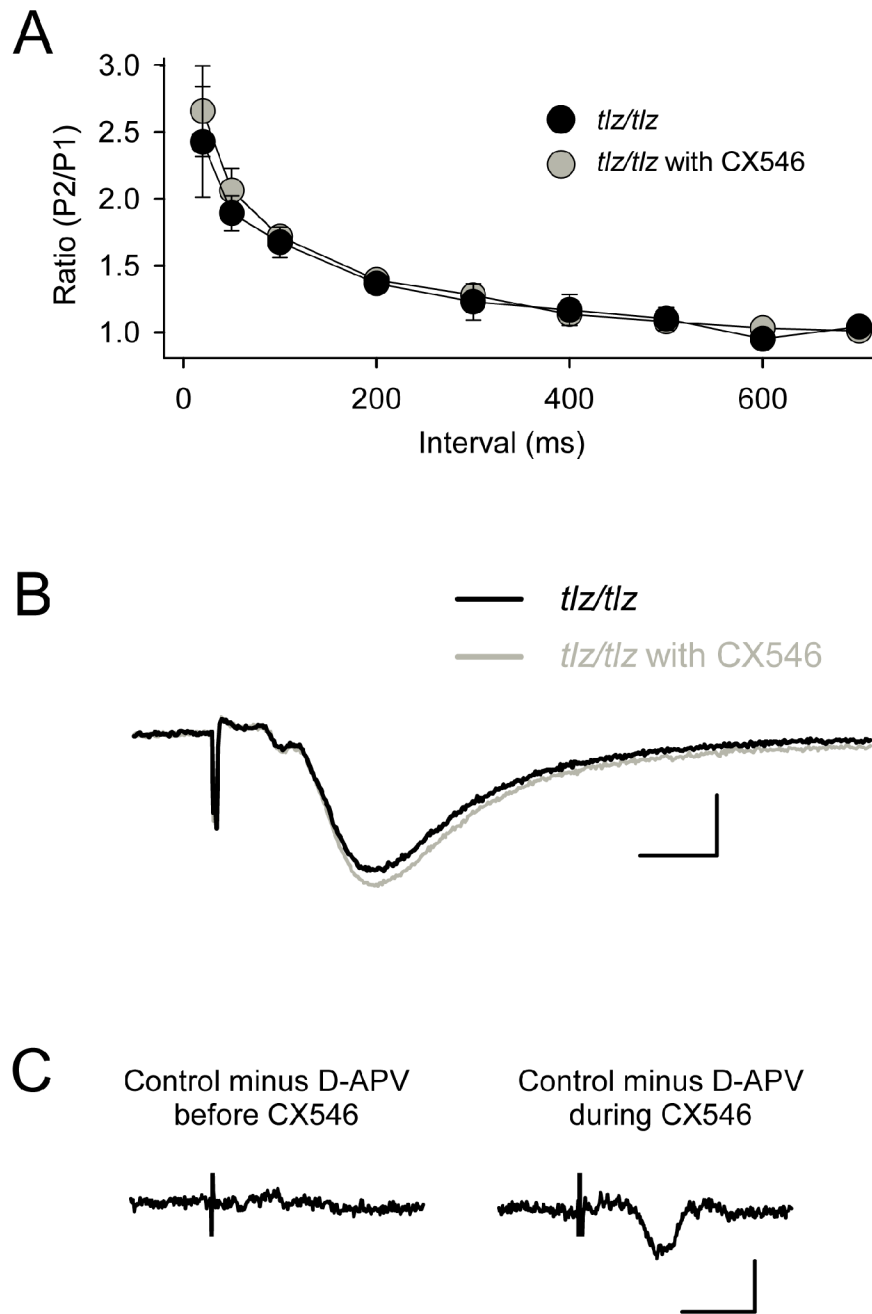

Supplement: Figure S10 — (A) Paired-pulse facilitation of fEPSPs in Neto1-null slices treated with (tlz/tlz with CX546; gray circles; n = 9) and without (tlz/tlz; black circles; n = 5) CX546 (25 μM). Interstimulus interval is indicated on the horizontal axis. P1, fEPSP slope first response; P2, fEPSP slope second response. (B) Representative traces show fEPSPs before (black trace) and 20–30 min after CX546 (25 μM; gray trace) administration in a hippocampal slice from a Neto1-null (tlz/tlz) mouse. Each fEPSP is the average of six consecutive traces. Scale bars: 5 ms, 0.2 mV. (C) Each trace shows the average difference plots before (n = 6 consecutive control fEPSPs) minus during D-APV (80 μM; n = 6 consecutive fEPSPs) from a single hippocampal slice from a Neto1-null mouse. Left trace was before administering CX546 and the right trace was during bath application of CX546 (25 μM). D-APV was washed out for 40 min before administering CX546. Scale bars: 10 ms, 0.1 mV. (150 KB PDF) [file pbio.1000041.sg010.pdf]

Figure S11

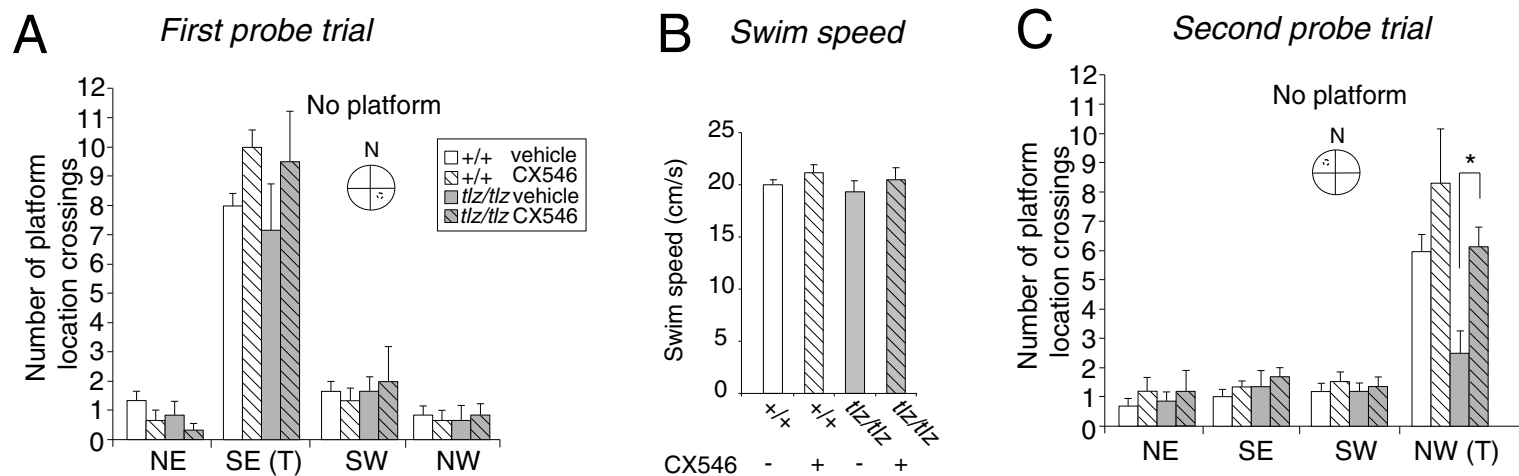

Supplement: Figure S11 — (A) Neto1+/+ and Neto1-null mice crossed the platform location with equal frequency in the first acquisition regardless of whether they were administered vehicle or CX546 (one-way ANOVA, F 3,20 = 1.78, p = 0.2). (B) Average swim speed was not different between Neto1+/+ and Neto1-null mice administered vehicle or 15 mg/kg CX546. Post hoc analysis did not indicate a difference in swim speed across groups (p > 0.6). (C) Neto1-null mice administered CX546 crossed the platform location in the second acquisition phase with equal frequency as compared to Neto1+/+ mice (p > 0.4). Neto1-null mice administered vehicle crossed the hidden platform location significantly fewer times compared with Neto1+/+ mice given vehicle (F 1,10 = 0.62; p < 0.05). Error bars represent ± standard error of the mean (SEM). (54 KB PDF) [file pbio.1000041.sg011.pdf]
