# Supplementary material for: Neto1 Is a Novel CUB-Domain NMDA Receptor–Interacting Protein Required for Synaptic Plasticity and Learning
Source: PLoS Biol. 2009 Feb 24;7(2):e1000041. doi: 10.1371/journal.pbio.1000041 (PMC2652390; doi:10.1371/journal.pbio.1000041)
Supplement: Table S1 — (13 KB PDF) [file pbio.1000041.st001.pdf]

Behavioural parameters in empty open field session

Parameters	*Neto1*$^{+/+}$	*Neto1*$^{tlz/tlz}$
Latency to escape the center, s	28.1 ± 6.0	33.8 ± 4.2
Central/wall activity, number of infrared beam breaks	0.35 ± 0.02	0.33 ± 0.04
Vertical activity, number of infrared beam breaks	54.3 ± 6.9	60.3 ± 3.2
Horizontal activity, number of infrared beam breaks	404.5 ± 15.9	384.5 ± 7.9
Risk assessment, number	5.7 ± 0.5	6.8 ± 0.9
Time freezing, s	30.8 ± 5.8	40.5 ± 3.2
Time grooming, s	24.9 ± 1.8	33.1 ± 3.4
