# Supplementary material for: Neto1 Is a Novel CUB-Domain NMDA Receptor–Interacting Protein Required for Synaptic Plasticity and Learning
Source: PLoS Biol. 2009 Feb 24;7(2):e1000041. doi: 10.1371/journal.pbio.1000041 (PMC2652390; doi:10.1371/journal.pbio.1000041)
Supplement: Table S2 — (47 KB PDF) [file pbio.1000041.st002.pdf]

Antibodies used in this study

Table S2

Antibody raised against	Host species	Source	IF Dilution	WB Dilution
NR2A	rabbit	Upstate (Cat. No. 07-632)	1:200	1:1,000
NR2A	rabbit	Molecular Probes (Cat. No. A-6473)		1:200
NR2B	rabbit	Upstate (Cat. No. 06-600)	1:200	
NR2B	rabbit	Novus (Cat. No. 300-106)		1:200
NeuN	mouse	Chemicon (Cat. No. MAB377)	1:2,000	
GluR2	rabbit	Chemicon (Cat. No. AB1506)		1:1,000
PSD-95	mouse	Affinity BioReagents (Cat. No. MA1-045)	1:200	1:2,000
PSD-95	rabbit	Synaptic Systems (Cat. No. 124002)	1:1,000	
Neto1	guinea pig	See materials and methods	1:100	1:300
Neto1	rabbit	See materials and methods		1:300
MAP2	rabbit	Chemicon (Cat. No. AB5622)	1:200	
MAP2	chicken	Chemicon (Cat. No. AB15452)	1:200	
NR1	mouse	BD Pharmingen (Cat. No. 556308)	1:25	1:500
HA	rabbit	Covance (Cat. No. PRB-101P)		1:1,000
HA	mouse	Covance (Cat. No. MMS-101R)		1:1,000
Vamp2	rabbit	Abcam (Cat. No. ab3347)		1:2,000
c-myc	mouse	Calbiochem (Cat. No. OP10)		1:500
Flag	mouse	Sigma (Cat. No. F3165)		1:4,000
β-actin	rabbit	Abcam (Cat. No. ab8227)		1:5,000
GABAaR alpha1	rabbit	Affinity BioReagents (Cat. No. OPA1-04100)		1:1,000
GFP	rabbit	Molecular Probes (Cat. No. A11122)		1:1,000
GFP	mouse	Molecular Probes (Cat. No. A11120)		1:1,000
β-gal	rabbit	ICN (Cat. No. 55976)		1:10,000

Secondary antibodies for immunofluoresence

anti-guinea pig Alexa-488	goat	Molecular Probes (Cat. No. A11073)	1:200	
anti-guinea pig Cy3	donkey	Jackson ImmunoResearch Laboratories (Cat. No. 706-166-148)	1:200	
anti-mouse Alexa-488	goat	Molecular Probes (Cat. No. A11029)	1:200	
anti-mouse Cy5	goat	Jackson ImmunoResearch Laboratories (Cat. No. 115-175-003)	1:200	
anti-rabbit Cy3	goat	Jackson ImmunoResearch Laboratories (Cat. No. 115-165-166)	1:200	
anti-rabbit Alexa-488	goat	Molecular Probes (Cat. No. A11034)	1:200	
anti-chicken Cy5	goat	Jackson ImmunoResearch Laboratories (Cat. No. 103-175-155)	1:200	

Secondary antibodies used for western blotting

anti-mouse HRP	goat	Sigma (Cat. No. A 4416)		1:5,000
anti-mouse HRP (TrueBlot)	not specified	eBioscience (Cat. No. 18-8877)		1:1,000
anti-guinea pig HRP	goat	Sigma (Cat. No. A 7289)		1:5,000
anti-rabbit HRP	goat	Jackson ImmunoResearch Laboratories (Cat. No. 111-035-144)		1:5,000
anti-rabbit HRP (TrueBlot)	not specified	eBioscience (Cat. No. 18-8816)		1:1,000
anti-rabbit HRP (light chain-specific)	mouse	Jackson ImmunoResearch Laboratories (Cat. No. 211-032-171)		1:5,000

Other

phalloidin-tetramethylrhodamine B isothiocyanate	n/a	Sigma (Cat. No. P 1951)	1:200	
